# Increased Frequency of Angiotensin‐Converting Enzyme D Allele in Asian Patients With Chronic Obstructive Pulmonary Disease: An Updated Meta‐Analysis

**DOI:** 10.1111/crj.70002

**Published:** 2024-08-26

**Authors:** Xiaozheng Wu, Wen Li, Zhenliang Luo, Yunzhi Chen

**Affiliations:** ^1^ Department of Preclinical Medicine Guizhou University of Traditional Chinese Medicine Guiyang China

**Keywords:** angiotensin‐converting enzyme, chronic obstructive pulmonary disease, COPD, meta‐analysis, polymorphism

## Abstract

At present, the angiotensin‐converting enzyme (ACE) I/D polymorphism was considered to be associated to the pathogenesis of chronic obstructive pulmonary disease (COPD). However, the association between it and the risk of COPD in different ethnic groups is still unclear. The purpose of this study is to conduct an updated meta‐analysis of the association between them; collect literatures published before 10 February 2023 by searching PubMed, Embase, MEDLINE, CBM, CNKI, Wanfang, and VIP Chinese scientific databases; and display the analysis results by drawing forest plots. At the same time, publication bias, sensitivity analysis, and trial sequential analysis (TSA) were performed to evaluate the stability and reliability of the results. In the overall population, the result of the DD versus II model showed the association with the risk of COPD ([OR] = 1.30, 95% CI [1.08, 1.56]), and there were no associations in other genetic models (*p* > 0.05). In Caucasians, the results of all genetic models showed no associations (*p* > 0.05). In Asians, the results of D versus I, DD versus II, and DD versus II + ID models showed the associations with the risk of COPD (D vs. I: [OR] = 1.48, 95% CI [1.14, 1.93]; DD vs. II: [OR] = 2.04, 95% CI [1.53, 2.72]; DD vs. II + ID: [OR] = 2.19, 95% CI [1.45, 3.29]), while the results of ID versus II and DD + ID versus II models showed no associations (*p* > 0.05). Therefore, the D allele and “DD” genotype variation of the ACE I/D gene polymorphism are associated with susceptibility to COPD in Asians but not in Caucasians.

## Graphical abstract

1. This study included data from case–control studies that have reported the association of ACE(I/D) polymorphism with COPD up to date with the aim of conducting an updated meta‐analysis of the relationship between ACE I/D gene polymorphisms and COPD susceptibility.
2. This study confirmed that the D allele and “DD” genotype variation of the ACE I/D gene polymorphism are associated with susceptibility to COPD in Asian populations but not in Caucasian populations.
3. Scientific and reliable statistical methods were used.

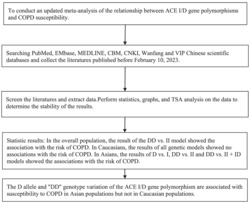

**Abbreviations**
95% CI: 95% confidence interval
ACE: angiotensin‐converting enzyme
Ang I: angiotensin I
Ang II: angiotensin II
ATS: American Thoracic Society
CBM: China Biology Medicine
CNKI: China National Knowledge Infrastructure
COPD: chronic obstructive pulmonary disease
D: deletion
ERS: European Respiratory Society
GOLD: Global Initiative for Chronic Obstructive Pulmonary Disease
HWE: Hardy–Weinberg equilibrium
I: insertion
NOS: Newcastle–Ottawa scale
OR: odds ratio
RIS: required information size
SNP: single nucleotide polymorphism
TSA: test sequence analysis

## 1 Introduction

The pathogenesis of chronic obstructive pulmonary disease (COPD) has been unclear until now, and the Global Initiative for Chronic Obstructive Pulmonary Disease in 2017 (GOLD 2017) pointed out that genetic factor was one of the important risk factors for COPD [1]. At present, there are many studies on the genetic susceptibility of COPD, and many candidate genes, including the angiotensin‐converting enzyme (ACE) gene, are considered to be related to the pathogenesis of COPD [2, 3].

The ACE gene is located on chromosome 17q23 (rs4646994), where the most important polymorphism is insertion/deletion (I/D) [4]. Studies had been conducted to determine whether the ACE gene was type I or D by the presence of DNA fragments consisting of 287 bases in intron 16 and Alu repeats [5]. Half of the ACE genes in the human body are regulated and controlled by I/D polymorphism. The I/D variants of the ACE gene can increase or decrease ACE activity in tissues and plasma [6, 7, 8, 9]. The function and activity of ACE will be altered if there is a single nucleotide polymorphism (SNP) I/D mutation in the ACE gene. Such I/D mutation can increase the level and activity of ACE, which will stimulate the development of pulmonary inflammation and lead to the onset of COPD [10, 11]. In addition, angiotensin II (Ang II) produced by the activation of ACE can not only promote the proliferation of blood vessels, cause vasoconstriction, increase blood pressure, and regulate water and salt metabolism (sodium retention and potassium excretion) but also is a strong proinflammatory factor [5, 12], which can also cause and aggravate COPD in the process of inflammation promotion.

There were meta‐analyses that found that D allele and DD genotype variants in the ACE polymorphism were the risk factors for COPD in Asian but not in Caucasian populations in the past years [13, 14]. However, in the last 4 years, there have been a number of new case–control studies examining the association between this polymorphism and the risk of COPD in different ethnic groups, with varying results. Thus, there are still no uniform conclusions about their association in different ethnic groups. Therefore, the present study conducted an updated meta‐analysis of their association to draw more objective and reliable conclusions.

## 2 Material and Methods [15]

### 2.1 Inclusion Criteria

(1) The case–control studies should conform to GOLD or the authoritative standards established by the American Thoracic Society/European Respiratory Society (ATS/ERS) or the Chinese Society of Respiratory Medicine, and the language of them is English or Chinese; (2) there was no restriction on the sex, age, race, and nationality of the cases; (3) the gene frequency data was complete and can be used to calculate the odds ratio (OR) and 95% confidence interval (95% CI), and the description of detection methods and means was accurate; (4) the distribution of genotype frequency of controls conformed to Hardy–Weinberg equilibrium (HWE) [16]; (5) the score of Newcastle–Ottawa scale (NOS) [17] was more than 7.

### 2.2 Exclusion Criteria

(1) Studies with incomplete gene frequency data were excluded; (2) studies of the type of reviews, in vivo and in vitro experiments, conference reports, and case reports were excluded; (3) family‐based studies were excluded; (4) for the same data being published multiple times, only one study with the most complete data was included.

### 2.3 Outcomes

The primary outcome is to assess the association of ACE (I/D) with the risk of COPD in the overall population.

The secondary outcome is to assess the association of ACE (I/D) with the risk of COPD in different populations and the differences in this association.

### 2.4 Retrieval Strategy

We searched using theme words and keywords combined with manual retrieval and literature tracking and collected literatures published before 10 February 2023 by searching PubMed, Embase, MEDLINE, China Biology Medicine (CBM), China National Knowledge Infrastructure (CNKI), Wanfang, and VIP Chinese scientific databases. The language was limited to English or Chinese. Search terms are “angiotensin converting enzyme” or “ACE” and “Chronic obstructive pulmonary disease” or “COPD” and “polymorphism.” See Table S1 in the supplemental content for details.

### 2.5 Literature Screening and Data Extraction

This work was first carried out independently by two researchers (Xiaozheng Wu and Wen Li). They initially screened the literatures against the inclusion and exclusion criteria, read the full text of the literatures that might meet the criteria, and then cross‐checked. If there was a disagreement between the parties during this process, the final decision will be made by a third party (Yunzhi Chen). If the data in the literatures was incomplete, try contacting the corresponding author of the literatures by email to obtain the most complete data possible. The extracted data included first author, year of publication, participants, country of the participants, ethnicity of the participants, diagnostic criteria, number of cases in each group, and frequency of each genotype.

### 2.6 Assessment of the Quality of Literature

The quality of all included literatures were assessed using NOS [17], with scores ranging from 0 to 9. If the score of the literature exceeded 7 points, it was considered to be of high quality.

### 2.7 Data Synthesis and Statistics

The study first used Pearson’s test to analyze the HWE of the control groups in SPSS24.0 and then analyzed all data in Revman 5.3 and Stata 14.0. Heterogeneity analyses were assessed by Q test and I^2^. If there was insignificant heterogeneity between studies (*p* > 0.1 or I^2^ < 50%), data were pooled using the fixed effects model to calculate OR and 95% CI. In contrast, the random effects model was used for pooled data analysis. All analysis results were visualized using forest plots, and funnel plots, Begg’s test, and Egger’s test were used to assess publication bias. If there was high heterogeneity between studies, sensitivity analysis would be conducted. In addition, test sequence analysis (TSA) was used to assess the stability of the results (set: [Type I error] probability = 5%, statistical test power = 80%, relative risk reduction = 20%).

## 3 Results

### 3.1 The Screening Results of Literatures

**FIGURE 1:**
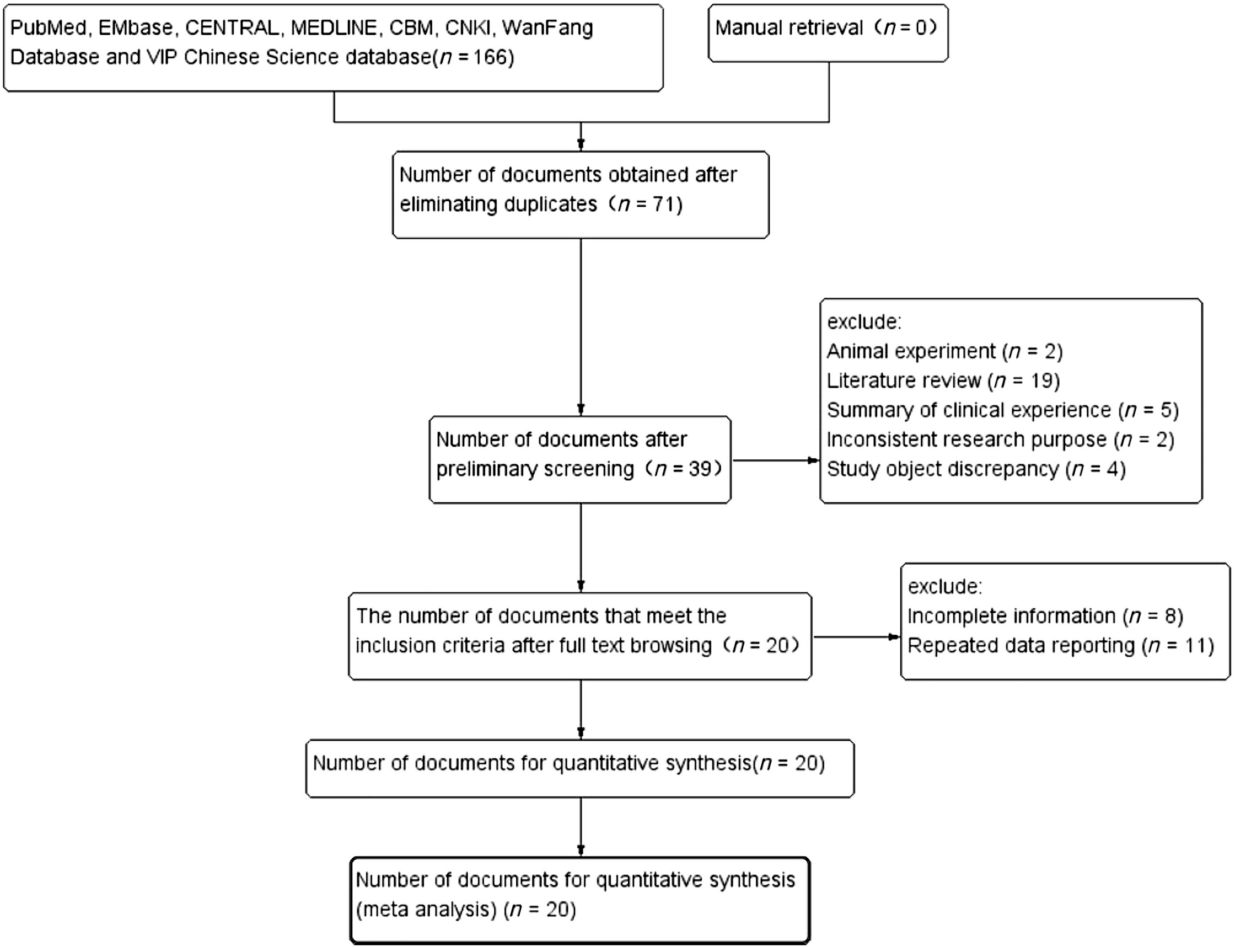
PRISMA literature screening flow chart.

One hundred sixty‐six relevant literatures were initially found in seven databases. And after rigorous screening, 20 studies (Caucasians: 12; Asians: eight) were finally included [5, 7, 8, 10, 11, 12, 18, 19, 20, 21, 22, 23, 24, 25, 26, 27, 28, 29, 30, 31], and they included 1730 COPD patients and 1930 healthy controls (Table 1/Table S2 in the supplemental content). The detailed flowchart (PRISMA statement [32]) is shown in Figure 1.

**TABLE 1:** Basic features of the included study (1).

Studies	Year	Country	Ethnicity	Diagnostic criteria	COPD (n)	Controls (n)	Gender (male/female) (n)		Age (years)	
							COPD	Controls	COPD	Controls
Ahsan [11]	2004	India	Asian	ATS/ERS	27	66	27/0	66/0	—	—
Ayada [18]	2014	Turkey	Caucasian	GOLD	47	64	—	—	—	—
Busquets [7]	2007	Spain	Caucasian	ATS/ERS	74	159	—	—	62 ± 2	—
Chen [19]	2014	China	Asian	GOLD	194	194	132/62	128/66	73.16 ± 9.8	72.7 ± 9.7
Gu [20]	2003	China	Asian	Chinese Society of Respiratory Medicine	122	159	75/47	81/78	60.8	63.5
Hopkinson [10]	2004	UK	Caucasian	GOLD	103	101	74/29	46/55	64.1 ± 9.1	61.8 ± 8.6
Jiang [21]	2002	China	Asian	Chinese Society of Respiratory Medicine	30	30	13/17	19/11	62.3 (34–78)	49.7 (35–78)
Kirtipal [22]	2019	India	Asian	ATS/ERS	200	200	134/66	114/86	52.1 ± 15.5	44.2 ± 14.4
Kuzubova [23]	2013	Russia	Caucasian	GOLD	63	95	63/0	95/0	60.4 ± 1.0	57.3 ± 1.7
Marushchak [24]	2019	Ukraine	Caucasian	ATS/ERS GOLD	25	20	—	—	—	—
Mlak [25]	2016	Poland	Caucasian	ATS/ERS GOLD	206	165	140/66	104/61	63 (32–85)	64 (29–92)
Pabst [26]	2009	Germany	Caucasian	ATS/ERS GOLD	152	158	104/48	62/96	62.8 ± 11.1	63.9 ± 18.4
Simsek [5]	2013	Turkey	Caucasian	GOLD	66	40	42/24	29/11	61.23 ± 11.41	59.70 ± 11.23
Tkacova [12]	2005	Slovakia	Caucasian	GOLD	66	118	49/17	80/38	65.4 ± 2.5	63.5 ± 1.2
Ulasli [27]	2013	Turkey	Caucasian	GOLD	50	49	47/3	39/19	64 ± 7.7	54.6 ± 7.8
van Suylen [28]	1999	Netherlands	Caucasian	ATS/ERS	87	95	62/25	42/53	65 (46–79)	50 (41–70)
Wang [29]	2000	China	Asian	Chinese Society of Respiratory Medicine	20	38	14/6	21/17	68.8 ± 2.1	52.1 ± 4.2
Wu [30]	2019	China	Asian	GOLD	95	82	55/40	52/30	73.51 ± 7.23	72.91 ± 7.30
Yildiz [31]	2003	Turkey	Caucasian	ATS/ERS	42	40	41/1	40/0	62 ± 7	60 ± 8
Zhang [8]	2008	China	Asian	ATS/ERS	61	57	51/10	44/13	63.0 ± 8.2	61.6 ± 7.3

Abbreviations: ATS: American Thoracic Society; COPD: chronic obstructive pulmonary disease; ERS: European Respiratory Society; GOLD: Global Initiative for Chronic Obstructive Lung Disease.

### 3.2 Quality Evaluation

All 20 studies had high NOS scores (≥7), indicating that they had a low risk of bias, as detailed in Table S3 in the supplemental content.

### 3.3 Meta‐Analysis

#### 3.3.1 Allele Genetic Model (D vs. I)

**FIGURE 2:**
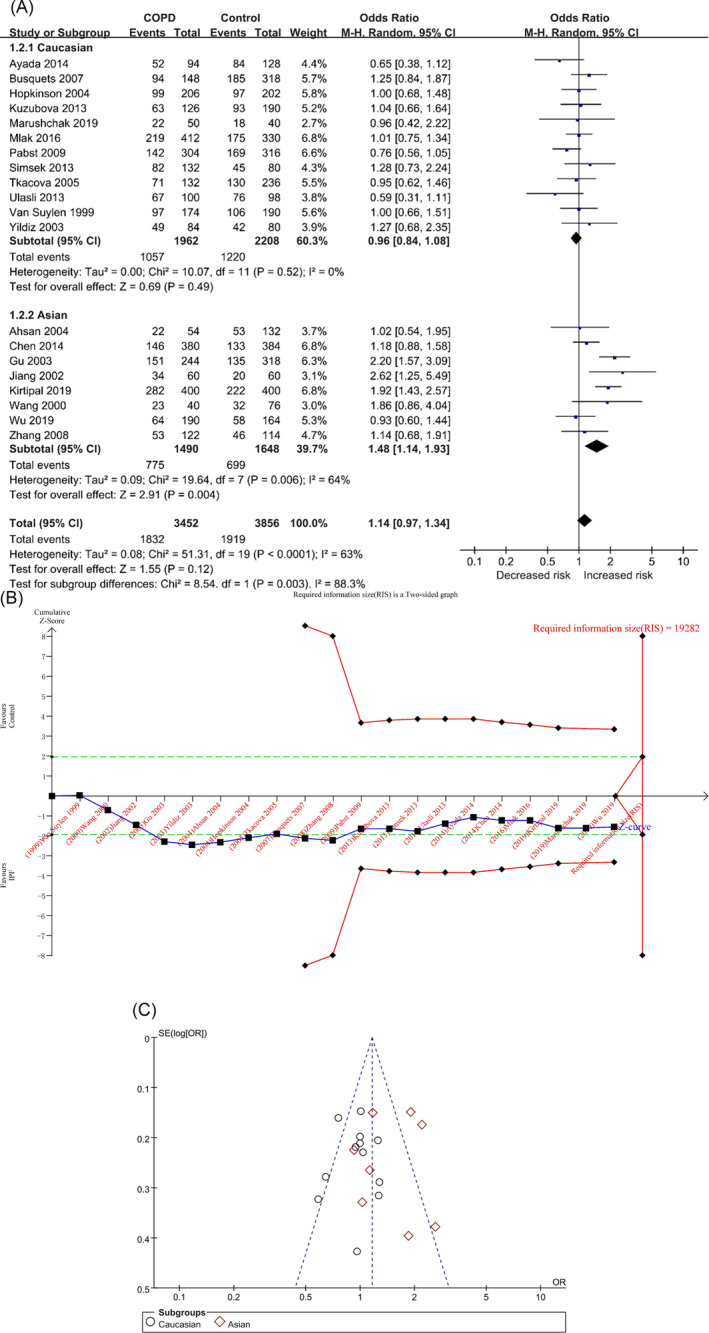
The D versus I model was used to evaluate the correlation between ACE gene polymorphism and COPD susceptibility. (A) The forest plot of the D versus I genetic model. (B) Trial sequential analysis of ACE polymorphism and COPD risk using the allelic model (D vs. I) (adjusted boundary print). *Note:* The combined sample size (*N* = 7308) did not exceed RIS (*N* = 19282), and the cumulative Z curve crossed the conventional boundary and did not cross the TSA boundary. (C) The funnel plot of the D versus I genetic model.

In the D vs. I model, the total number of cases in COPD and control groups after pooled studies were 3452 and 3856, respectively. The heterogeneity analysis showed that *p* < 0.0001 and I^2^ = 63%, and the D allele was not associated with the risk of COPD in the overall population ([OR] = 1.14, 95% CI [0.97, 1.34], *p* = 0.12) (Table 2 and Figure 2A). In the TSA, the combined sample size did not exceed the required information size (RIS) (RIS = 19282), and the cumulative Z curve crossed the conventional boundary but did not cross the TSA boundary, indicating that more studies need to be included to verify the results of meta‐analysis (Figure 2B/Figure S1 in the supplemental content). Sensitivity analysis showed that the two studies, Gu, Zhang, and Yang [20] and Kirtipal, Thakur, and Sobti [22], had certain sensitivity (Figure S2/Table S4 in the supplemental content). The funnel plot appeared to be asymmetrical (Figure 2C), but the results of Begg’s and Egger’s tests showed that there was no obvious bias (P_Begg_ = 0.719; P_Egger_ = 0.907) (Table 2/Table S5 and Figures S3 and S4 in the supplemental content).

**TABLE 2:** The results of meta‐analysis and publication bias.

Genetic model	Subgroup	Study (n)	Heterogeneity test		Sample		Model	OR [95% CI]	Effect *p* value	Publication bias	
			*p* values	I^2^ (%)	COPD (n)	Control (n)				P_Begg_	P_Egger_
D vs. I	Caucasian	12	0.52	0	1962	2208	Fixed	0.96 [0.84, 1.08]	0.49	0.681	0.982
	Asian	8	0.006	64	1490	1648	Random	1.48 [1.14, 1.93]	0.004	1.000	0.851
	Total	20	< 0.0001	63	3452	3856	Random	1.14 [0.97, 1.34]	0.12	0.719	0.907
DD vs. II	Caucasian	12	0.65	0	522	580	Fixed	0.92 [0.72, 1.18]	0.52	0.784	0.756
	Asian	8	0.19	30	478	433	Fixed	2.04 [1.53, 2.72]	< 0.00001	0.621	0.879
	Total	20	0.01	46	1000	1013	Fixed	1.30 [1.08, 1.56]	0.006	0.631	0.865
ID vs. II	Caucasian	12	0.79	0	682	756	Fixed	0.97 [0.78, 1.22]	0.81	0.411	0.494
	Asian	8	0.39	6	491	670	Fixed	0.89 [0.70, 1.13]	0.34	0.216	0.652
	Total	20	0.73	0	1173	1426	Fixed	0.93 [0.79, 1.10]	0.40	0.905	0.750
DD + ID vs. II	Caucasian	12	0.79	0	981	1104	Fixed	0.95 [0.77, 1.17]	0.63	0.273	0.775
	Asian	8	0.48	0	749	826	Fixed	1.21 [0.97, 1.50]	0.09	0.621	0.523
	Total	20	0.65	0	1730	1930	Fixed	1.07 [0.92, 1.24]	0.41	0.472	0.857
DD vs. II + ID	Caucasian	12	0.43	1	981	1104	Fixed	0.94 [0.78, 1.14]	0.53	0.493	0.571
	Asian	8	0.03	55	749	826	Random	2.19 [1.45, 3.29]	0.0002	0.805	0.337
	Total	20	< 0.00001	69	1730	1930	Random	1.31 [0.98, 1.74]	0.07	0.472	0.839

In each subgroup, the heterogeneity analysis showed (Table 2 and Figure 2A) Caucasians (*p* = 0.52, I^2^ = 0%) and Asians (*p* = 0.006, I^2^ = 64%), and the D allele was associated with the risk of COPD in Asians but not in Caucasians: Caucasians ([OR] = 0.96, 95% CI [0.84, 1.08], *p* = 0.49) and Asians ([OR] = 1.48, 95% CI [1.14, 1.93], *p* = 0.004) (Table 2 and Figure 2A). In the TSA of Caucasian population, the combined sample size did not exceed the RIS (RIS = 6429), and the cumulative Z curve did not cross the traditional boundary and TSA boundary (Figures S5 and S6 in the supplementary content). In the TSA of Asian population, the combined sample size did not exceed the RIS (RIS = 21206), and the cumulative Z curve crossed the traditional boundary but did not cross the TSA boundary (Figures S7 and S8 in the supplemental content). It shows that they all need to include more studies to verify the results. The funnel plots of the two populations were not very symmetrical (Figures S9 and S10 in the supplemental content), but the results of Begg’s and Egger’s tests showed that there was no obvious bias (Caucasians: P_Begg_ = 0.681, P_Egger_ = 0.982; Asians: P_Begg_ = 1.000, P_Egger_ = 0.851) (Table 2/Table S5 in the supplemental content).

#### 3.3.2 Additive Genetic Model (DD vs. II)

**FIGURE 3:**
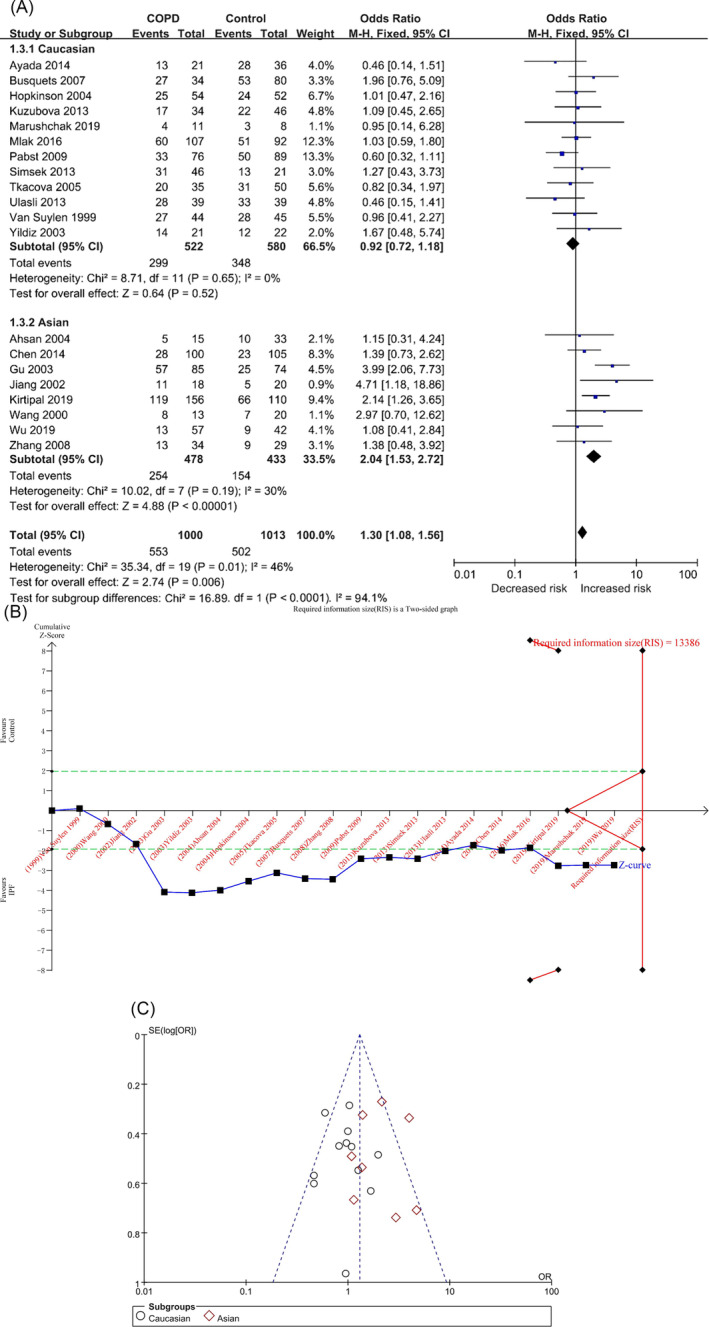
The DD versus II model was used to evaluate the correlation between ACE gene polymorphism and COPD susceptibility. (A) The forest plot of DD versus II genetic model. (B) Trial sequential analysis of ACE polymorphism and COPD risk using the additive genetic model (DD vs. II) (adjusted boundary print). *Note:* The combined sample size (*N* = 2013) did not exceed RIS (*N* = 13386), and the cumulative Z curve crossed the conventional boundary and did not cross the TSA boundary. (C) The funnel plot of the DD versus II genetic model.

In the DD versus II model, there were 1000 and 1013 cases in the COPD and control groups, respectively, and there was no significant heterogeneity among the studies (*p* = 0.01, I^2^ = 46%). The DD genotype was associated with the risk of COPD in the overall population ([OR] = 1.30, 95% CI [1.08, 1.56], *p* = 0.006) (Table 2 and Figure 3A), and the result of TSA was similar to the D versus I model (Figure 3B/Figure S11 in the supplemental content). Sensitivity analysis showed that the two studies, Gu, Zhang, and Yang [20] and Kirtipal, Thakur, and Sobti [22], had certain sensitivity (Figure S12/Table S6 in the supplemental content). The funnel plot appeared to be asymmetrical (Figure 3C), but the results of Begg’s and Egger’s tests showed that there was no obvious bias (P_Begg_ = 0.631; P_Egger_ = 0.865) (Table 2/Table S7 and Figures S13 and S14 in the supplemental content).

In each subgroup, the heterogeneity analysis showed (Table 2 and Figure 3A) Caucasians (*p* = 0.65, I^2^ = 0%) and Asians (*p* = 0.19, I^2^ = 30%), and the DD genotype was associated with the risk of COPD in Asians but not in Caucasians: Caucasians ([OR] = 0.92, 95% CI [0.72, 1.18], *p* = 0.52) and Asians ([OR] = 2.04, 95% CI [1.53, 2.72], *p* < 0.00001) (Table 2 and Figure 3A). The results of TSA of the two populations were similar to the D versus I model (Figures S15–S18 in the supplemental content). The funnel plots of the two populations were not very symmetrical (Figures S19 and S20 in the supplemental content), but the results of Begg’s and Egger’s tests showed that there was no obvious bias (Caucasians: P_Begg_ = 0.784, P_Egger_ = 0.756; Asians: P_Begg_ = 0.621, P_Egger_ = 0.879) (Table 2/Table S7 in the supplemental content).

#### 3.3.3 Heterozygous Genetic Model (ID vs. II)

**FIGURE 4:**
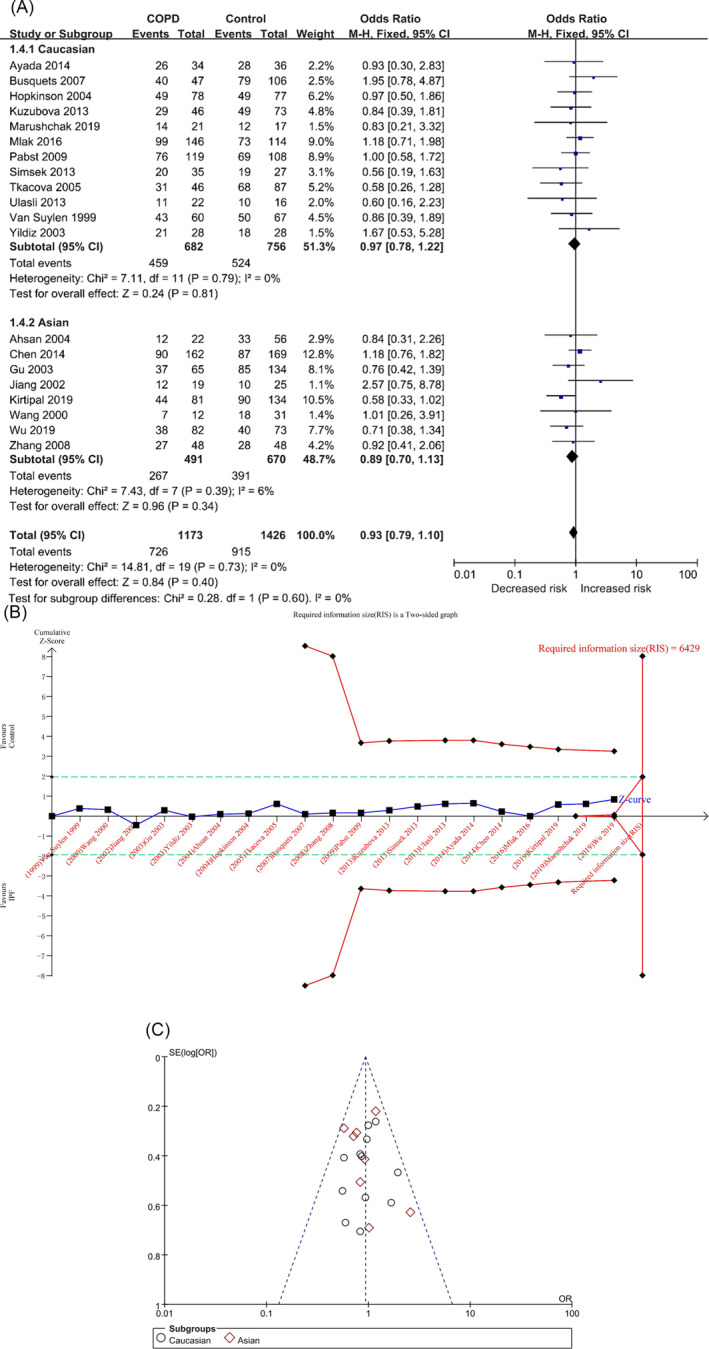
The ID versus II model was used to evaluate the correlation between ACE gene polymorphism and COPD susceptibility. (A) The forest plot of ID versus II genetic model. (B) Trial sequential analysis of ACE polymorphism and COPD risk using the heterozygous genetic model (ID vs. II) (adjusted boundary print). *Note:* The combined sample size (*N* = 2599) did not exceed RIS (*N* = 6429), and the cumulative Z curve did not cross the conventional boundary and the TSA boundary. (C) The funnel plot of the ID versus II genetic model.

In the ID versus II model, there were 1173 and 1426 cases in the COPD and control groups, respectively, and there was no significant heterogeneity among the studies (*p* = 0.73, I^2^ = 0%). The result showed that there was no association between them in the overall population ([OR] = 0.93, 95% CI [0.79, 1.10], *p* = 0.40) (Table 2 and Figure 4A), and the results of TSA were similar to the D versus I model of Caucasian population (Figure 4B/Figure S21 in the supplemental content). Sensitivity analysis showed no significant sensitivity in each study (Table S8 and Figure S22 in the supplemental content). The funnel plot appeared to be asymmetrical (Figure 4C), but the results of Begg’s and Egger’s tests showed that there was no obvious bias (P_Begg_ = 0.905; P_Egger_ = 0.750) (Table 2/Table S9 and Figures S23 and S24 in the supplemental content).

In each subgroup, the heterogeneity analysis showed (Table 2 and Figure 4A) Caucasians (*p* = 0.79, I^2^ = 0%) and Asians (*p* = 0.39, I^2^ = 6%), and the ID genotype was not associated with the risk of COPD in Caucasians and Asians: Caucasians ([OR] = 0.97, 95% CI [0.78, 1.22], *p* = 0.81) and Asians ([OR] = 0.89, 95% CI [0.70, 1.13], *p* = 0.34) (Table 2 and Figure 4A). The results of TSA were similar to the ID versus II model of the overall population (Figures S25–S28 in the supplemental content). The funnel plots of the two populations are not very symmetrical (Figures S29 and S30 in the supplemental content), but the results of Begg’s and Egger’s tests showed that there was no obvious bias (Caucasians: P_Begg_ = 0.411, P_Egger_ = 0.494; Asians: P_Begg_ = 0.216, P_Egger_ = 0.652) (Table 2/Table S9 in the supplemental content).

#### 3.3.4 Dominant Genetic Model (DD + ID vs. II)

**FIGURE 5:**
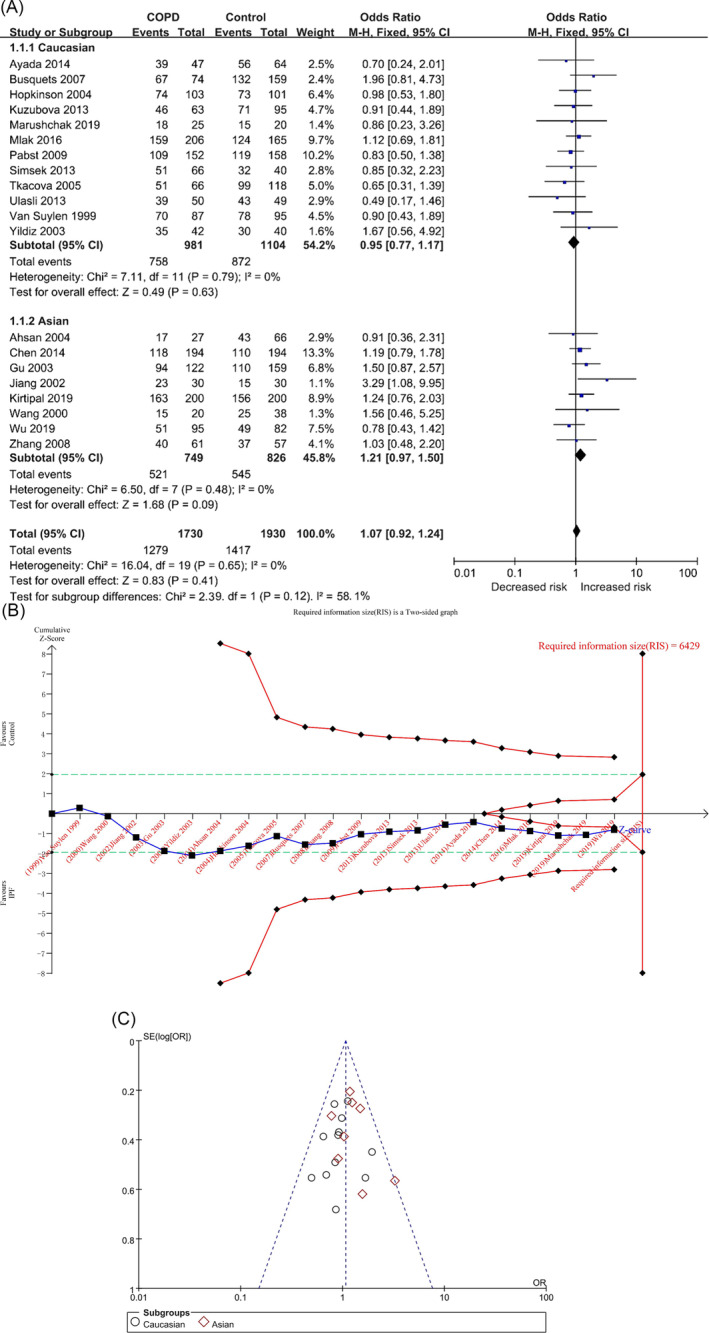
The DD + ID versus II model was used to evaluate the correlation between ACE gene polymorphism and COPD susceptibility. (A) The forest plot of the DD + ID versus II genetic model. (B) Trial sequential analysis of ACE polymorphism and COPD risk using the dominant genetic model (DD + ID vs. II) (adjusted boundary print). *Note:* The combined sample size (*N* = 3660) did not exceed RIS (*N* = 6429), and the cumulative Z curve crossed the conventional boundary and did not cross the TSA boundary. (C) The funnel plot of the DD + ID versus II genetic model.

In the DD + ID versus II model, there were 1730 and 1930 cases in the COPD and control groups, respectively, and there was no significant heterogeneity among the studies (*p* = 0.65, I^2^ = 0%). The DD + ID genotype was not associated with the risk of COPD in the overall population ([OR] =1.07, 95% CI [0.92, 1.24], *p* = 0.41) (Table 2 and Figure 5A), and the result of TSA was similar to the D versus I model (Figure 5B/Figure S31) in supplemental content). Sensitivity analysis showed no significant sensitivity in each study (Table S10 and Figure S32 in the supplemental content). The funnel plot appeared to be asymmetrical (Figure 5C), but the results of Begg’s and Egger’s tests showed that there was no bias (P_Begg_ = 0.472; P_Egger_ = 0.857) (Table 2/Table S11 and Figures S33 and S34 in the supplemental content).

In each subgroup, the heterogeneity analysis showed (Table 2, Figure 5A) Caucasians (*p* = 0.79, I^2^ = 0%) and Asians (*p* = 0.48, I^2^ = 0%), and the DD + ID genotype was not associated with the risk of COPD in Caucasians and Asians: Caucasians ([OR] = 0.95, 95% CI [0.77, 1.17], *p* = 0.63) and Asians ([OR] = 1.21, 95% CI [0.97, 1.50], *p* = 0.09) (Table 2 and Figure 5A). The results of TSA of the two populations were similar to the D versus I model (Figures S35–S38 in the supplemental content). The funnel plots of the two populations were not very symmetrical (Figures S39 and S40 in the supplemental content), but the results of Begg’s and Egger’s tests showed that there was no obvious bias (Caucasians: P_Begg_ = 0.273, P_Egger_ = 0.775; Asians: P_Begg_ = 0.621, P_Egger_ = 0.523) (Table 2/Table S11 in the supplemental content).

#### 3.3.5 Recessive Genetic Model (DD vs. II + ID)

**FIGURE 6:**
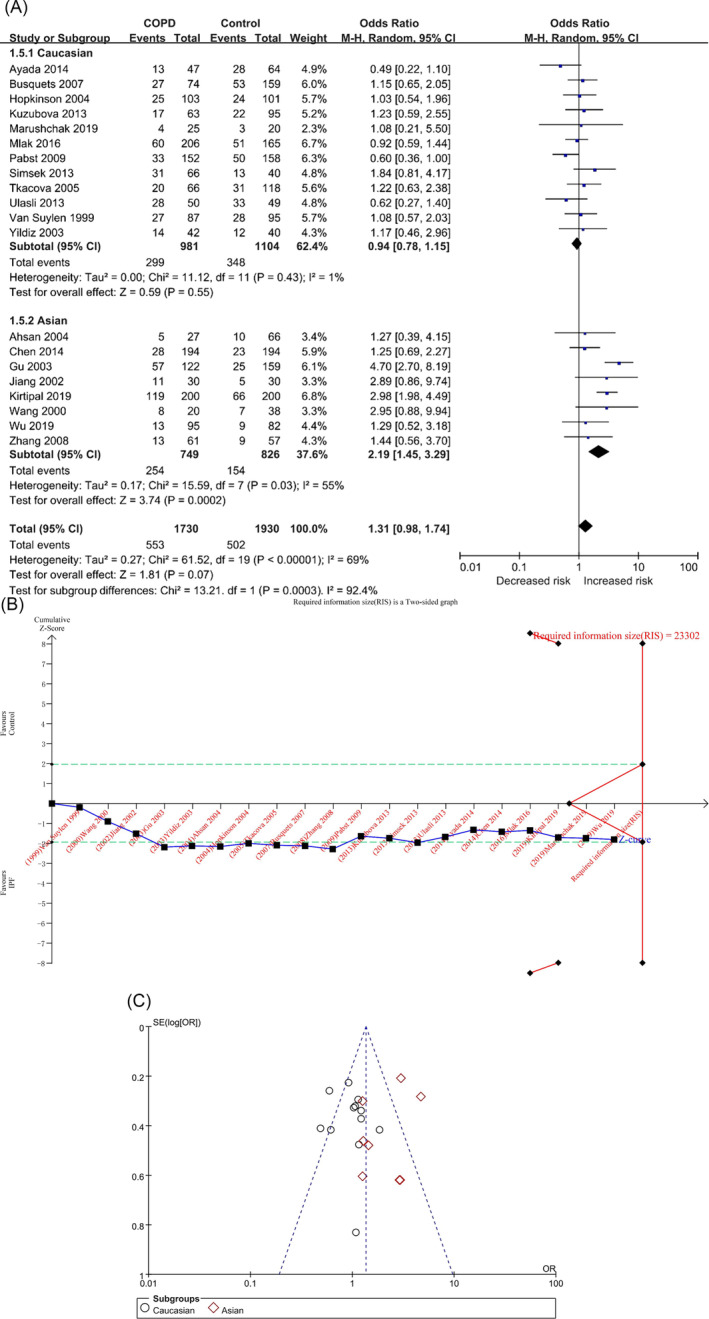
The DD versus II + ID model was used to evaluate the correlation between ACE gene polymorphism and COPD susceptibility. (A) The forest plot of DD versus II + ID genetic model. (B) Trial sequential analysis of ACE polymorphism and COPD risk using the recessive genetic model (DD vs. II + ID) (adjusted boundary print). *Note:* The combined sample size (*N* = 3660) exceeded RIS (*N* = 23302), and the cumulative Z curve crossed the conventional boundary and did not cross the TSA boundary. (C) The funnel plot of the DD versus II + ID genetic model.

In the DD versus II + ID model, there were 1730 and 1930 cases in the COPD and control groups, respectively, and there was significant heterogeneity among the studies (*p* < 0.00001, I^2^ = 69%). The DD genotype was not associated with the risk of COPD in the overall population ([OR] = 1.31, 95% CI [0.98, 1.74], *p* = 0.07) (Table 2 and Figure 6A), and the result of TSA was similar to the D versus I model (Figure 6B/Figure S41 in the supplemental content). Sensitivity analysis showed that the two studies, Gu, Zhang, and Yang [20] and Kirtipal, Thakur, and Sobti [22], had a certain sensitivity (Table S12 and Figure S42 in the supplemental content). The funnel plot appeared to be asymmetrical (Figure 6C), but the results of Begg’s and Egger’s tests showed that there was no obvious bias (P_Begg_ = 0.472; P_Egger_ = 0.839) (Table 2/Table S13 and Figures S43 and S44 in the supplemental content).

In each subgroup, the heterogeneity analysis showed (Table 2 and Figure 6A) Caucasians (*p* = 0.43, I^2^ = 1%) and Asians (*p* = 0.03, I^2^ = 55%). The DD genotype was associated with the risk of COPD in Asians but not in Caucasians: Caucasians ([OR] = 0.94, 95% CI [0.78, 1.14], *p* = 0.53) and Asians ([OR] = 2.19, 95% CI [1.45, 3.29], *p* = 0.0002) (Table 2 and Figure 6A). The results of TSA of the two populations were similar to the D versus I model (Figures S45–S48 in the supplemental content). The funnel plots of the two populations were not very symmetrical (Figures S49 and S50 in the supplemental content), but the results of Begg’s and Egger’s tests showed that there was no obvious bias (Caucasians: P_Begg_ = 0.493, P_Egger_ = 0.571; Asians: P_Begg_ = 0.805, P_Egger_ = 0.337) (Table 2/Table S13 in the supplemental content).

### 3.4 Heterogeneity

#### 3.4.1 D versus I

The result of the D versus I model showed that there is heterogeneity. After sensitivity analysis, we found that two studies [20, 22] in the Asian population were sensitive, which proves that the heterogeneity of the D versus I model may come from them. Therefore, we used three methods to verify the heterogeneity and sensitivity of the D versus I model: (1) The result showed no statistical change in the Asian population (fixed: *p* < 0.00001) when the statistical analysis model of combined effect size was adjusted, but there was a change in the overall population (fixed: *p* = 0.002); (2) the results showed that the effect size of the D versus I model in the Asian population changed significantly (OR: *p* = 0.006, I^2^ = 64%; RR: *p* = 0.07, I^2^ = 46%) when the research method with the largest weight was adjusted, but there was no change on statistical significance (OR: *p* = 0.004; RR: *p* = 0.0005), and the results for the overall population did not change (OR: *p* < 0.0001, I^2^ = 63%; RR: *p* = 0.0001, I^2^ = 63%) (OR: *p* = 0.12; RR: *p* = 0.13); (3) the included studies were excluded one by one, and the effect size and *p* changes were observed. The results showed that the D versus I model had *p* = 0.02 (no change) in Asians and *p* = 0.26 (no change) in the overall population, and the effect size did not change significantly (Asian: *p* = 0.03, I^2^ = 57%; overall: *p* = 0.006, I^2^ = 51%) when the literature of Gu, Zhang, and Yang [20] was excluded, which proved that Gu, Zhang, and Yang [20] was not the main source of heterogeneity. The results were the same after excluding Kirtipal, Thakur, and Sobti [22] (Asian: *p* = 0.03; overall: *p* = 0.25) (Asian: *p* = 0.01, I^2^ = 63%; overall: *p* = 0.003, I^2^ = 53%). Through the analysis of these three methods, it is confirmed that the results of the D vs. I model were unstable. In addition, it is also proved that Gu, Zhang, and Yang [20] and Kirtipal, Thakur, and Sobti [22] were not the main sources of heterogeneity of the D versus I model, and the heterogeneity might come from different detection methods in the studies of the D versus I model, so the reported gene frequencies were different.

#### 3.4.2 DD versus II + ID

The result of the DD versus II + ID model showed that there was heterogeneity. After sensitivity analysis, we found that two studies [20, 22] in the Asian population were sensitive, which proved that the heterogeneity of the DD versus II + ID model may come from them. Therefore, we have used the same three methods in the D versus I model described above to verify its heterogeneity and sensitivity: (1) Method 1: The result showed no statistical change in the Asian population (fixed: *p* < 0.00001), but there was a change in the overall population (fixed: *p* < 0.0001); (2) Method 2: The results showed that the effect size of the DD versus II + ID model in the Asian population changed significantly (OR: *p* = 0.03, I^2^ = 55%; RR: *p* = 0.17, I^2^ = 32%), but there was no change on statistical significance (OR: *p* = 0.0002; RR: *p* < 0.00001), and the results for the overall population did not change (OR: *p* < 0.00001, I^2^ = 69%; RR: *p* < 0.00001, I^2^ = 67%) (OR: *p* = 0.07; RR: *p* = 0.07); (3) Method 3: The results showed that the DD versus II + ID model had *p* = 0.02 (no change) in Asians and *p* = 0.26 (no change) in the overall population, and the effect size changed significantly in Asians but not in the overall population (Asian: *p* = 0.19, I^2^ = 31%; overall: *p* = 0.002, I^2^ = 56%) when the literature of Gu, Zhang, and Yang [20] was excluded, which proved that Gu, Zhang, and Yang [20] may be the main source of heterogeneity in Asians but not in the overall population. There was no effect on the results after excluding Kirtipal, Thakur, and Sobti [22] (Asian: *p* = 0.007; overall: *p* = 0.15) (Asian: *p* = 0.03, I^2^ = 57%; overall: *p* = 0.0005, I^2^ = 60%). Through the analysis of these three methods, it is confirmed that the results of the DD versus II + ID model were unstable. In addition, it was proved that Gu, Zhang, and Yang [20] were the main source of heterogeneity rather than Kirtipal, Thakur, and Sobti [22]. However, this heterogeneity had no impact on the meta‐analysis results of Asians and may have a certain impact on the results of the overall population.

#### 3.4.3 DD versus II

There is no obvious heterogeneity in the DD vs. II model, but after sensitivity analysis, we found that in the DD vs. II model, two studies [20, 22] in the Asian population had a certain sensitivity. Therefore, we carried out the three methods here again to verify the heterogeneity and sensitivity of the DD vs. II model: (1) Method 1: The result showed no statistical change in Asians (random: *p* = 0.0002) but showed a change in the overall population (random: *p* = 0.09); (2) Method 2: The results showed that the effect size of the DD versus II model did not change significantly (OR: *p* = 0.19, I^2^ = 30%; RR: *p* = 0.32, I^2^ = 14%), and there was no change on statistical significance in Asians (OR: *p* < 0.00001; RR: *p* < 0.00001). Moreover, the results for the overall population did not change (OR: *p* = 0.01, I^2^ = 46%; RR: *p* = 0.02, I^2^ = 44%) (OR: *p* = 0.006; RR: *p* = 0.006); (3) Method 3: The results showed that the DD versus II model had *p* = 0.0007 (no change) in Asians and *p* = 0.12 (changed) in the overall population, and the effect size did not change significantly in the Asian population and in the overall population (Asian: *p* = 0.53, I^2^ = 0%; overall: *p* = 0.18, I^2^ = 22%) when the literature of Gu, Zhang, and Yang [20] was excluded, which proved that Gu, Zhang, and Yang [20] may be the main source of heterogeneity in the overall population but not in Asians. The same result appeared after excluding Kirtipal, Thakur, and Sobti [22] (Asian: *p* < 0.0001 [no change]; overall: *p* = 0.06 [changed]) (Asian: *p* = 0.13, I^2^ = 40% [no change]; overall: *p* = 0.03, I^2^ = 43% [no change]). It was proved that there was instability of the results of the DD versus II model after taking the three methods to analyze. And it was confirmed that Gu, Zhang, and Yang [20] and Kirtipal, Thakur, and Sobti [22] had no heterogeneity, and their sensitivity had no effect on the results of the Asian population, which mainly affected the results of the overall population.

## 4 Discussion

The pathogenesis of COPD is not yet particularly clear. However, in recent years, the ACE gene variant has been reported to be associated with the pathogenesis of COPD [2, 3]. ACE is a zinc‐containing hydroxyl ectohydrolase that cleaves low‐activity angiotensin I (Ang I) to the highly active 8‐peptide Ang II, which is a potent vasoconstrictor and inflammatory factor [33, 34, 35]. Previous studies had found that changes in ACE gene expression in plasma were strongly associated with the onset of a variety of lung diseases [36]. The I/D is the most important polymorphism in the ACE gene, it is the I or D of 287 bp DNA fragments in intron 16, and the increased frequency of D allele can increase the content and expression of ACE in serum [36]. In the past, meta‐analyses had found that the variation of D allele and DD genotype in it was a risk factor for COPD in Asians, but not in Caucasians [13, 14]. However, in the recent 4 years, there had been many new studies on the association between it and the risk of COPD in different ethnic groups, and the results of these studies were different. Therefore, there are still no uniform conclusions about their association in different ethnic groups.

This work included 20 case–control studies conducted in Caucasians and Asians, including 1730 patients with COPD and 1930 controls, and five genetic models were used to evaluate the association between ACE (I/D) polymorphisms and the risk of COPD. In the overall population, the heterogeneity analysis found that the D versus I model and the DD versus II + ID model were heterogeneous, so the random effect model was used to evaluate them, and the rest of the genetic models were evaluated by using the fixed effects model. The result of the DD versus II model showed that there was an association with the risk of COPD, and the other genetic models showed no association, indicating that people with the “DD” genotype may have the risk of COPD.

In subgroup analysis, heterogeneity analysis found that there was no heterogeneity in all genetic models of the Caucasian population, so the fixed effect model was used to evaluate them. The results of all genetic models showed no association with the risk of COPD in Caucasians, which was the same as that of recent studies [24, 25]. For the Asian population, heterogeneity analysis found that there was heterogeneity in the D versus I model and the DD versus II + ID model, so the random effect model was used to evaluate them, and other genetic models were evaluated by the fixed effect model. The results of D versus I, DD versus II, and DD versus II + ID models showed the association with the risk of COPD, but the analysis results of ID versus II and DD + ID versus II models showed no association with the risk of COPD, indicating that people carrying the D allele and the “DD” genotype are at risk of developing COPD in Asians. These results are similar to the results of two recent studies [22, 37]. The funnel plots showed none of them to be symmetrical, but the Begg’s and Egger’s tests showed that there were no obvious biases. Therefore, these results are stable and reliable.

Due to the heterogeneity of the D versus I model and the DD versus II + ID model, and the sensitivity of Gu, Zhang, and Yang [20] and Kirtipal, Thakur, and Sobti [22] of D versus I, DD versus II + ID, and DD versus II models in the Asian population, we verified the heterogeneity and sensitivity of these three models. Except that Gu, Zhang, and Yang [20] were the main source of heterogeneity of the DD versus II + ID model, and Gu, Zhang, and Yang [20] and Kirtipal, Thakur, and Sobti [22] were not the main sources of heterogeneity of the D versus I model and the DD versus II model. Moreover, Gu, Zhang, and Yang [20] and Kirtipal, Thakur, and Sobti [22] had no impact on the results of the Asian population but had a certain impact on the results of the overall population. Therefore, the results of the three models of D versus I, DD versus II + ID, and DD versus II are relatively stable in the Asian population, and the sensitivity of Gu, Zhang, and Yang [20] and Kirtipal, Thakur, and Sobti [22] has no effect on the results of the Asian population. This sensitivity mainly affects the results of D versus I, DD versus II + ID, and DD versus II models in the overall population.

## 5 Limitations

Firstly, heterogeneity affects the reliability of results. Although we carefully analyzed and verified the sources of heterogeneity and sensitivity of D versus I and DD versus II + ID as well as the sources of sensitivity of DD versus II, we found that the heterogeneity and sensitivity mainly affected the results of the overall population, not the results of Asian population, but the presence of such heterogeneity and sensitivity may affect the reliability of the results for the Asian population. Secondly, the small sample size may also affect the reliability of the results. The TSA results of the overall and Asian populations showed that the combined sample size of all genetic models did not exceed the RIS, and the cumulative Z‐curves only crossed traditional boundaries (ID versus II model did not), but none of them crossed the TSA boundary; in the Caucasian population, the combined sample size of all genetic models did not exceed the RIS, and the cumulative Z‐curves did not cross the traditional boundaries and the TSA boundary. All these results indicate insufficient sample size, which inevitably leads to bias and some false negative results. Thirdly, the ethnicities in the study are limited. This study only collected data on Caucasian and Asian populations and did not have genetic data on African and mixed populations. In addition, the interaction between genes and the environment was not documented in detail in all the original literatures, so further analysis of the interaction between them is not possible.

## 6 Conclusions

This study confirms that the D allele and “DD” genotype of ACE I/D gene polymorphism are associated with susceptibility to COPD in Asians, but not Caucasians, based on previous studies. But considering the limitations, it is necessary to include more high‐quality case–control studies with large samples for analysis in order to obtain more reliable results in the future.

## Author Contributions

This study is initiated by Xiaozheng Wu. Xiaozheng Wu will develop the search strategies, conduct data collection, and analyze it independently. Zhenliang Luo, Wen Li, and Yunzhi Chen will revise it. All authors have approved the final manuscript. Conceptualization: Xiaozheng Wu. Methodology: Xiaozheng Wu and Wen Li. Software: Xiaozheng Wu. Supervision: Yunzhi Chen. Writing – original draft: Xiaozheng Wu. Writing – review and editing: Zhenliang Luo, Wen Li, and Yunzhi Chen.

## Ethics Statement

This review does not require ethical approval because the included studies are published data and do not involve the patients’ privacy. The results of this review will be reported in accordance with the PRISMA extension statement and disseminated to a peer‐reviewed journal.

## Conflicts of Interest

The authors declare no conflicts of interest.

## Supporting information


**Figure S1** Trial sequential analysis of ACE polymorphism and COPD risk using the allelic model (D vs. I) (adjusted boundary sketch).
**Figure S2** Influence analysis results of D versus I
**Figure S3** D versus I funnel chart generated by Begg’s test.
**Figure S4** D versus I funnel chart of bias generation detected by Egger’s test.
**Figure S5** Trial sequential analysis of ACE polymorphism and COPD risk in Caucasian using the allelic model (D vs. I) (adjusted boundary print).
**Figure S6** Trial sequential analysis of ACE polymorphism and COPD risk in Caucasian using the allelic model (D vs. I) (adjusted boundary sketch).
**Figure S7** Trial sequential analysis of ACE polymorphism and COPD risk in Asian using the allelic model (D vs. I) (adjusted boundary print).
**Figure S8** Trial sequential analysis of ACE polymorphism and COPD risk in Asian using the allelic model (D vs. I) (adjusted boundary sketch).
**Figure S9** Inverted funnel chart of D versus I of Caucasian.
**Figure S10** Inverted funnel chart of D versus I of Asian.
**Figure S11** Trial sequential analysis of ACE polymorphism and COPD risk using the additive genetic model (DD vs. II) (adjusted boundary sketch).
**Figure S12** Influence analysis results of DD versus II.
**Figure S13** DD versus II funnel chart generated by Begg’s test.
**Figure S14** DD versus II funnel chart of bias generation detected by Egger’s test.
**Figure S15** Trial sequential analysis of ACE polymorphism and COPD risk in Caucasian using the additive genetic model (DD vs. II) (adjusted boundary print).
**Figure S16** Trial sequential analysis of ACE polymorphism and COPD risk in Caucasian using the additive genetic model (DD vs. II) (adjusted boundary sketch).
**Figure S17** Trial sequential analysis of ACE polymorphism and COPD risk in Asian using the additive genetic model (DD vs. II) (adjusted boundaries print). D versus I.
**Figure S18** Trial sequential analysis of ACE polymorphism and COPD risk in Asian using the additive genetic model (DD vs. II) (adjusted boundary sketch).
**Figure S19** Inverted funnel chart of DD versus II of Caucasian.
**Figure S20** Inverted funnel chart of DD versus II of Asian. ID versus II.
**Figure S21** Trial sequential analysis of ACE polymorphism and COPD risk using the heterozygous genetic model (ID vs. II) (adjusted boundary sketch).
**Figure S22** Influence analysis results of ID versus II.
**Figure S23** ID versus II funnel chart generated by Begg’s test.
**Figure S24** ID versus II funnel chart of bias generation detected by Egger’s test.
**Figure S25** Trial sequential analysis of ACE polymorphism and COPD risk in Caucasian using the heterozygous genetic model (ID vs. II) (adjusted boundary print).
**Figure S26** Trial sequential analysis of ACE polymorphism and COPD risk in Caucasian using the heterozygous genetic model (ID vs. II) (adjusted boundary sketch).
**Figure S27** Trial sequential analysis of ACE polymorphism and COPD risk in Asian using the heterozygous genetic model (ID vs. II) (adjusted boundary print).
**Figure S28** Trial sequential analysis of ACE polymorphism and COPD risk in Asian using the heterozygous genetic model (ID vs. II) (adjusted boundary sketch).
**Figure S29** Inverted funnel chart of ID versus II of Caucasian.
**Figure S30** Inverted funnel chart of ID versus II of Asian. DD + ID versus II.
**Figure S31** Trial sequential analysis of ACE polymorphism and COPD risk using the dominant genetic model (DD + ID vs. II) (adjusted boundary sketch).
**Figure S32** Influence analysis results of DD + ID versus II.
**Figure S33** DD + ID versus II funnel chart generated by Begg’s test.
**Figure S34** DD + ID versus II funnel chart of bias generation detected by Egger’s test.
**Figure S35** Trial sequential analysis of ACE polymorphism and COPD risk in Caucasian using the dominant genetic model (DD + ID vs. II) (adjusted boundary print).
**Figure S36** Trial sequential analysis of ACE polymorphism and COPD risk in Caucasian using the dominant genetic model (DD + ID vs. II) (adjusted boundary sketch).
**Figure S37** Trial sequential analysis of ACE polymorphism and COPD risk in Asian using the dominant genetic model (DD + ID vs. II) (adjusted boundary print).
**Figure S38** Trial sequential analysis of ACE polymorphism and COPD risk in Asian using the dominant genetic model (DD + ID vs. II) (adjusted boundary sketch).
**Figure S39** Inverted funnel chart of DD + ID versus II of Caucasian.
**Figure S40** Inverted funnel chart of DD + ID versus II of Asian. DD versus II + ID.
**Figure S41** Trial sequential analysis of ACE polymorphism and COPD risk using the recessive genetic model (DD vs. II + ID) (adjusted boundary sketch).
**Figure S42** Influence analysis results of DD versus II + ID.
**Figure S43** DD versus II + ID funnel chart generated by Begg’s test.
**Figure S44** DD versus II + ID funnel chart of bias generation detected by Egger’s test.
**Figure S45** Trial sequential analysis of ACE polymorphism and COPD risk in Caucasian using the recessive genetic model (DD vs. II + ID) (adjusted Boundary print).
**Figure S46** Trial sequential analysis of ACE polymorphism and COPD risk in Caucasian using the recessive genetic model (DD vs. II + ID) (adjusted boundary sketch).
**Figure S47** Trial sequential analysis of ACE polymorphism and COPD risk in Asian using the recessive genetic model (DD vs. II + ID) (adjusted boundary print).
**Figure S48** Trial sequential analysis of ACE polymorphism and COPD risk in Asian using the recessive genetic model (DD vs. II + ID) (adjusted boundary sketch).
**Figure S49** Inverted funnel chart of DD versus II + ID of Caucasian.
**Figure S50** Inverted funnel chart of DD versus II + ID of Asian.
**Table S1** PubMed search strategy.
**Table S2** Basic features of the included study (2).
**Table S3** Newcastle–Ottawa scale (NOS).
**Table S4** Influence analysis results of D versus I.
**Table S5** Results of Begg’s test and Egger’s test to detect D versus I bias in different populations.
**Table S6** Influence analysis results of DD versus II.
**Table S7** Results of Begg’s test and Egger’s test to detect DD versus II bias in different populations.
**Table S8** Influence analysis results of ID versus II.
**Table S9** Results of Begg’s test and Egger’s test to detect ID versus II bias in different populations.
**Table S10** Influence analysis results of DD + ID versus II.
**Table S11** Results of Begg’s test and Egger’s test to detect DD + ID versus II bias in different populations.
**Table S12** Influence analysis results of DD versus II + ID.
**Table S13** Results of Begg’s test and Egger’s test to detect DD versus II + ID bias in different populations.

## Data Availability

The data that support the findings of this study are available from the corresponding author upon reasonable request.
