# Supplementary material for: Increased Frequency of Angiotensin‐Converting Enzyme D Allele in Asian Patients With Chronic Obstructive Pulmonary Disease: An Updated Meta‐Analysis
Source: Clin Respir J. 2024 Aug 26;18(8):e70002. doi: 10.1111/crj.70002 (PMC11347616; doi:10.1111/crj.70002)
Supplement: Supplementary file 1 — Figure S1 Trial sequential analysis of ACE polymorphism and COPD risk using the allelic model (D vs. I) (adjusted boundary sketch). Figure S2 Influence analysis results of D versus I Figure S3 D versus I funnel chart generated by Begg’s test. Figure S4 D versus I funnel chart of bias generation detected by Egger’s test. Figure S5 Trial sequential analysis of ACE polymorphism and COPD risk in Caucasian using the allelic model (D vs. I) (adjusted boundary print). Figure S6 Trial sequential analysis of ACE polymorphism and COPD risk in Caucasian using the allelic model (D vs. I) (adjusted boundary sketch). Figure S7 Trial sequential analysis of ACE polymorphism and COPD risk in Asian using the allelic model (D vs. I) (adjusted boundary print). Figure S8 Trial sequential analysis of ACE polymorphism and COPD risk in Asian using the allelic model (D vs. I) (adjusted boundary sketch). Figure S9 Inverted funnel chart of D versus I of Caucasian. Figure S10 Inverted funnel chart of D versus I of Asian. Figure S11 Trial sequential analysis of ACE polymorphism and COPD risk using the additive genetic model (DD vs. II) (adjusted boundary sketch). Figure S12 Influence analysis results of DD versus II. Figure S13 DD versus II funnel chart generated by Begg’s test. Figure S14 DD versus II funnel chart of bias generation detected by Egger’s test. Figure S15 Trial sequential analysis of ACE polymorphism and COPD risk in Caucasian using the additive genetic model (DD vs. II) (adjusted boundary print). Figure S16 Trial sequential analysis of ACE polymorphism and COPD risk in Caucasian using the additive genetic model (DD vs. II) (adjusted boundary sketch). Figure S17 Trial sequential analysis of ACE polymorphism and COPD risk in Asian using the additive genetic model (DD vs. II) (adjusted boundaries print). D versus I. Figure S18 Trial sequential analysis of ACE polymorphism and COPD risk in Asian using the additive genetic model (DD vs. II) (adjusted boundary sketch). Figure S19 Inve [file CRJ-18-e70002-s001.docx]

**D vs.I**

**
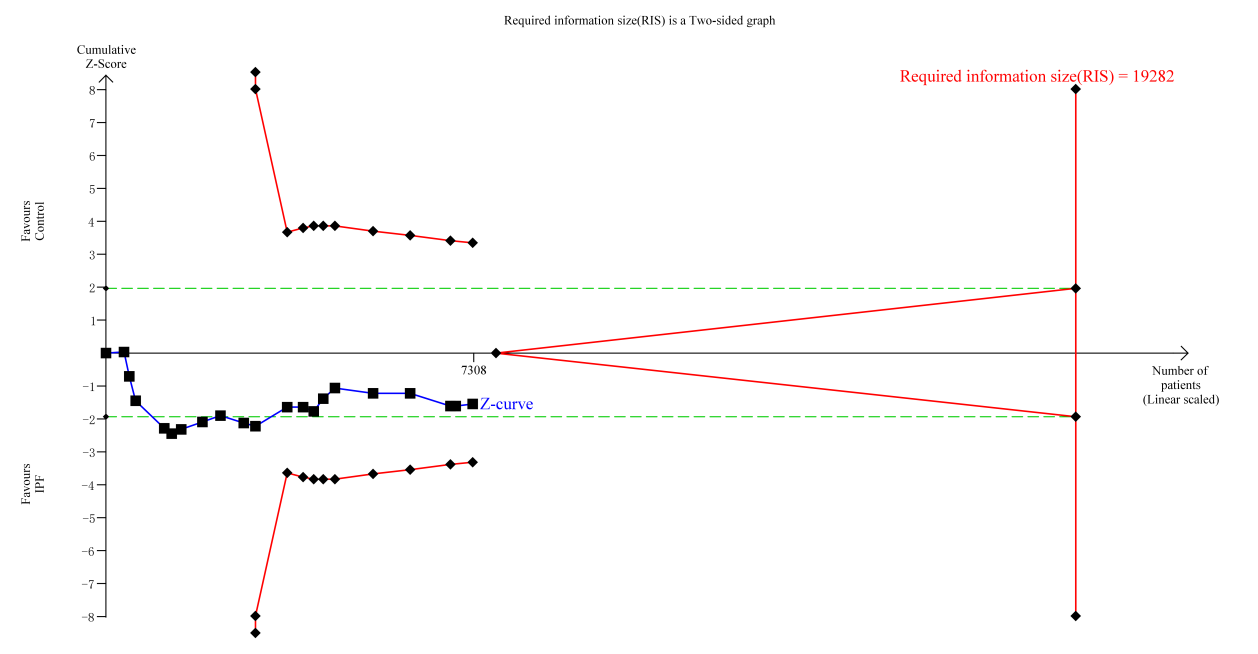
**

**Figure S1 Trial sequential analysis of ACE polymorphism and COPD risk using the allelic model (D vs.I)(Adjusted Boundaries Sketch).**

**Note:The combined sample size(N=7308) did not exceed RIS(N=19282),and the cumulative Z curve crossed the conventional boundary and did not cross the TSA boundary.**

**
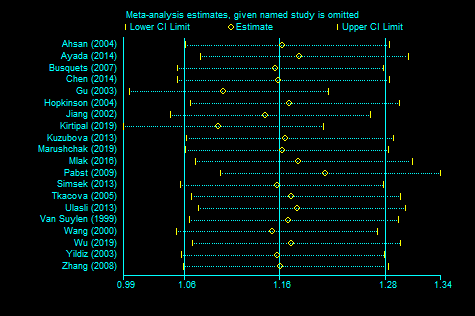
**

**Figure S2 Influence analysis results of D vs.I**

**
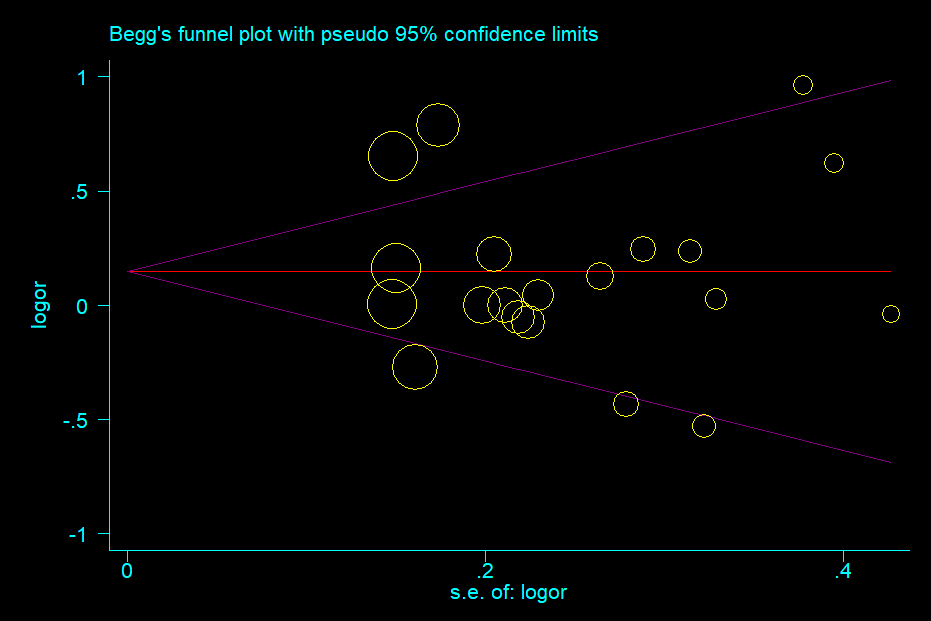
**

**Figure S3 D vs.I funnel chart generated by Begg's Test**

**
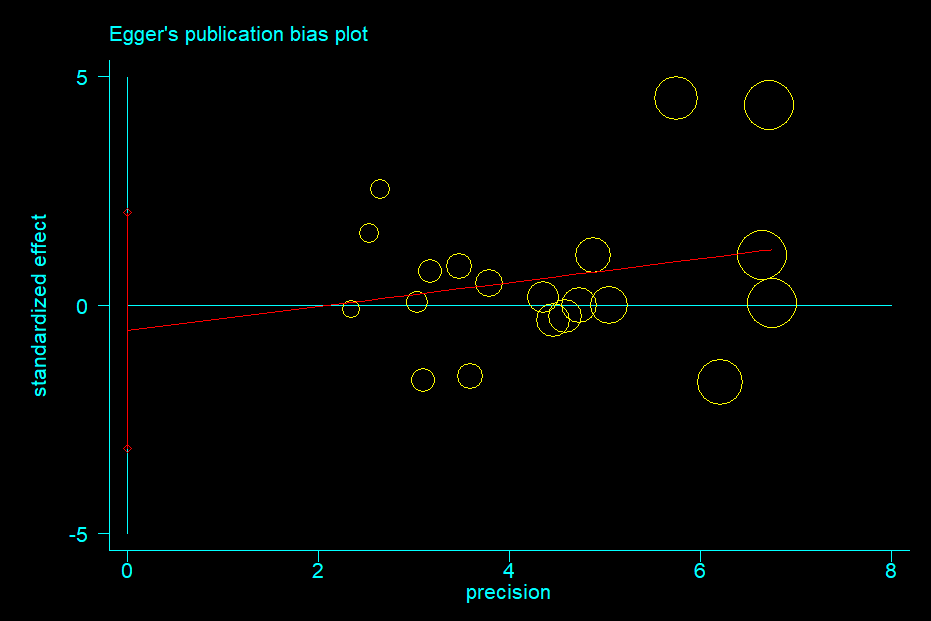
**

**Figure S4 D vs.I funnel chart of bias generation detected by Egger's test**

**
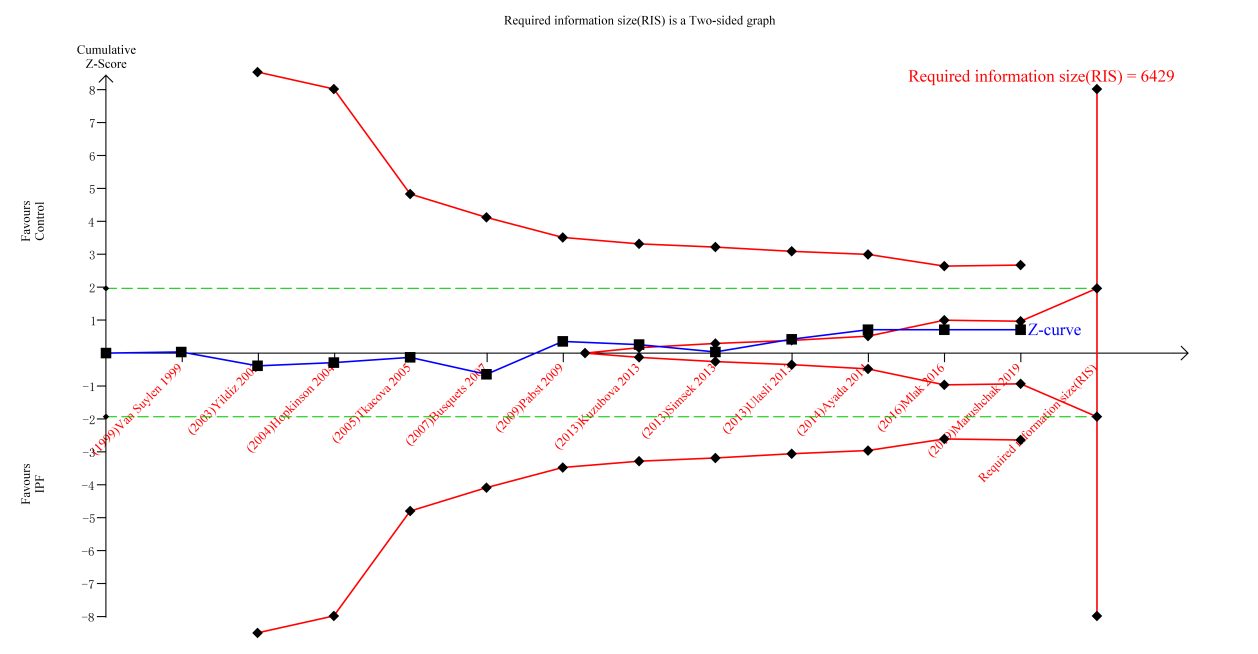
**

**Figure S5 Trial sequential analysis of ACE polymorphism and COPD risk in Caucasian using the allelic model (D vs.I)(Adjusted Boundaries Print)**

**
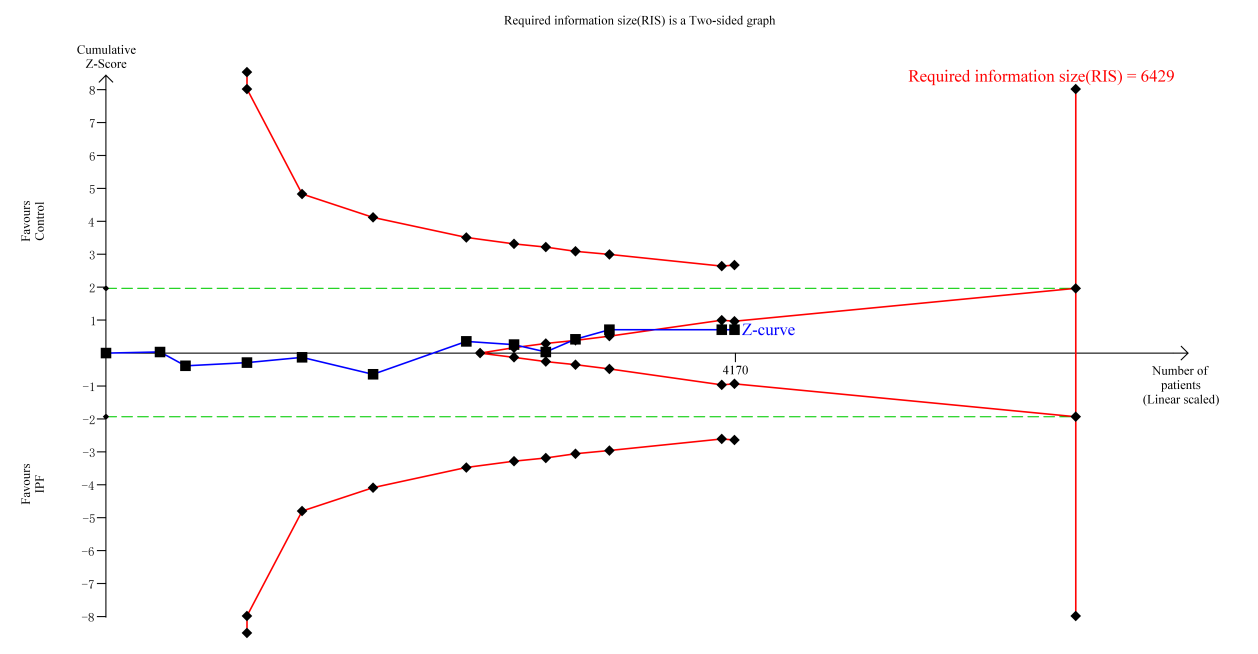
**

**Figure S6 Trial sequential analysis of ACE polymorphism and COPD risk in Caucasian using the allelic model (D vs.I)(Adjusted Boundaries Sketch)**

**
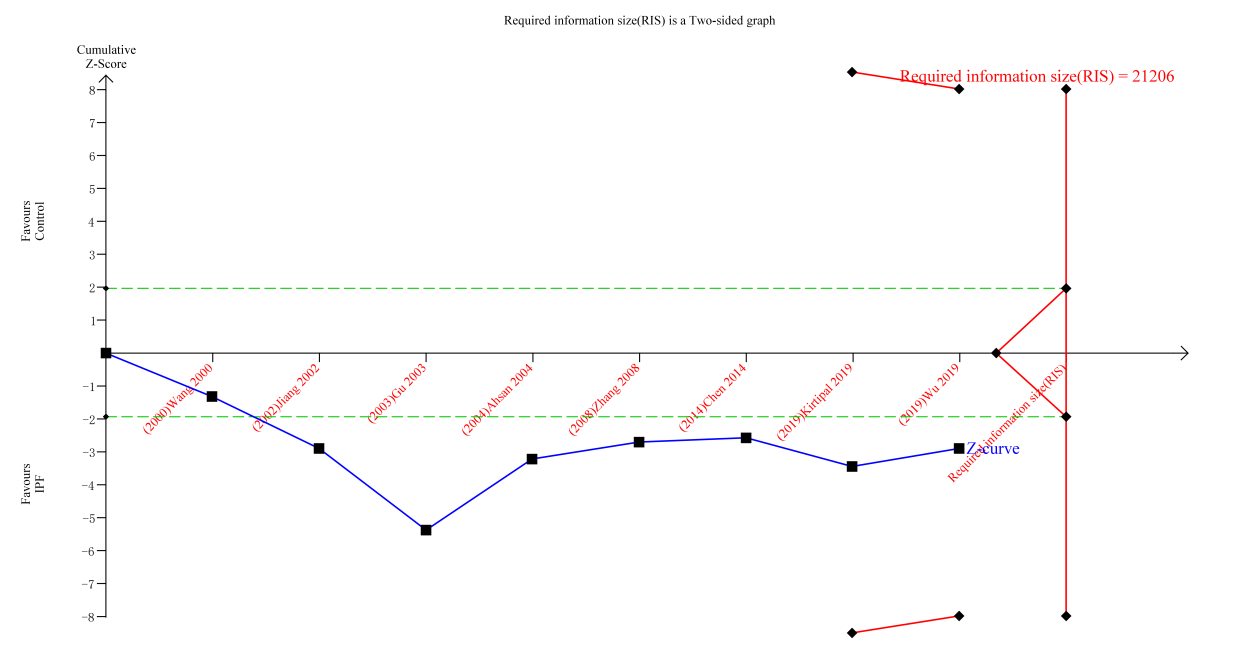
**

**Figure S7 Trial sequential analysis of ACE polymorphism and COPD risk in Asian using the allelic model (D vs.I)(Adjusted Boundaries Print)**

**
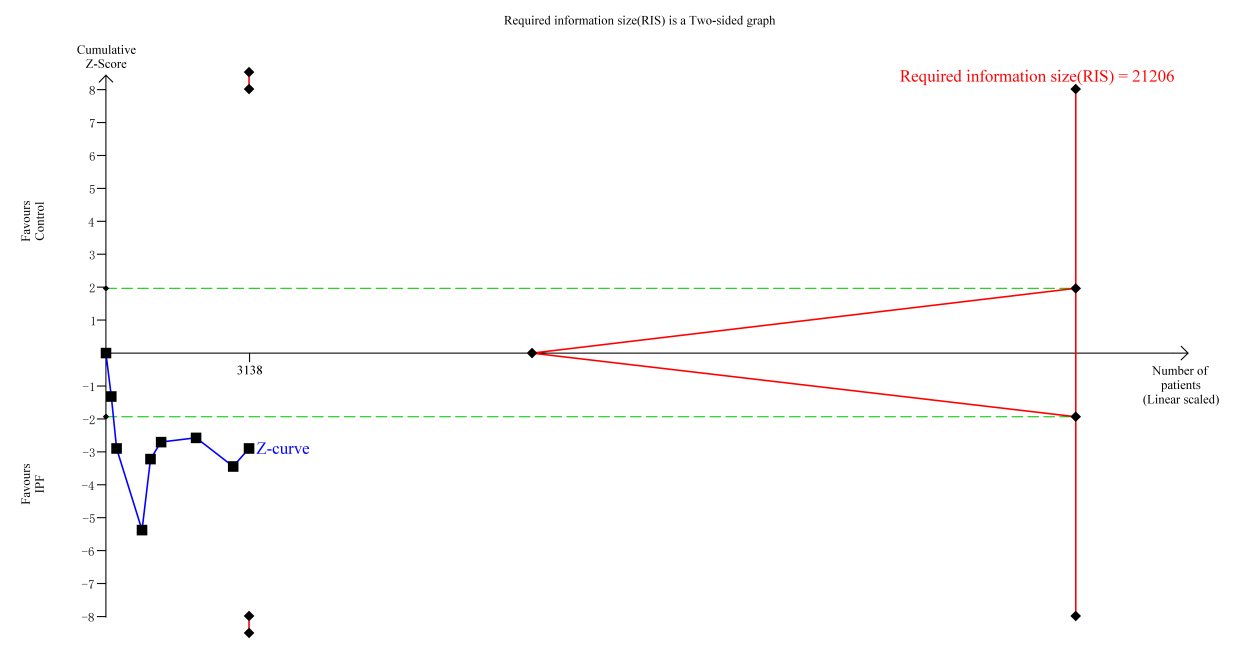
**

**Figure S8 Trial sequential analysis of ACE polymorphism and COPD risk in Asian using the allelic model (D vs.I)(Adjusted Boundaries Sketch)**

**
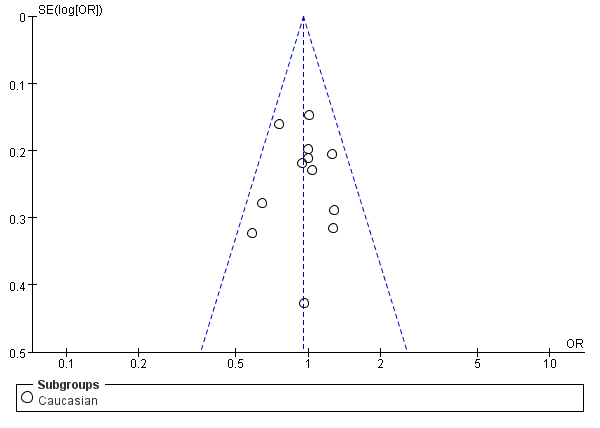
**

**Figure S9 Inverted funnel chart of D vs.I of Caucasian**

**
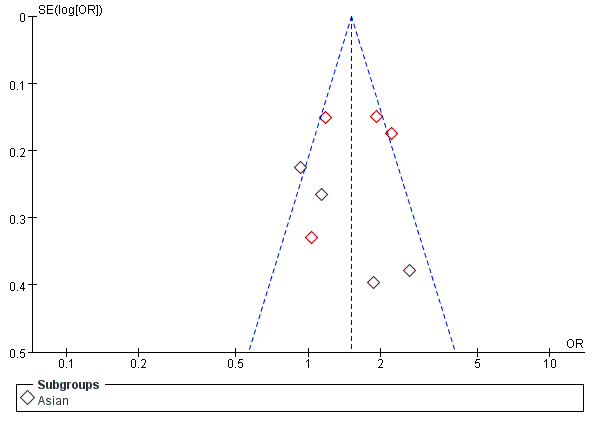
**

**Figure S10 Inverted funnel chart of D vs.I of Asian**

**DD vs. II**

**
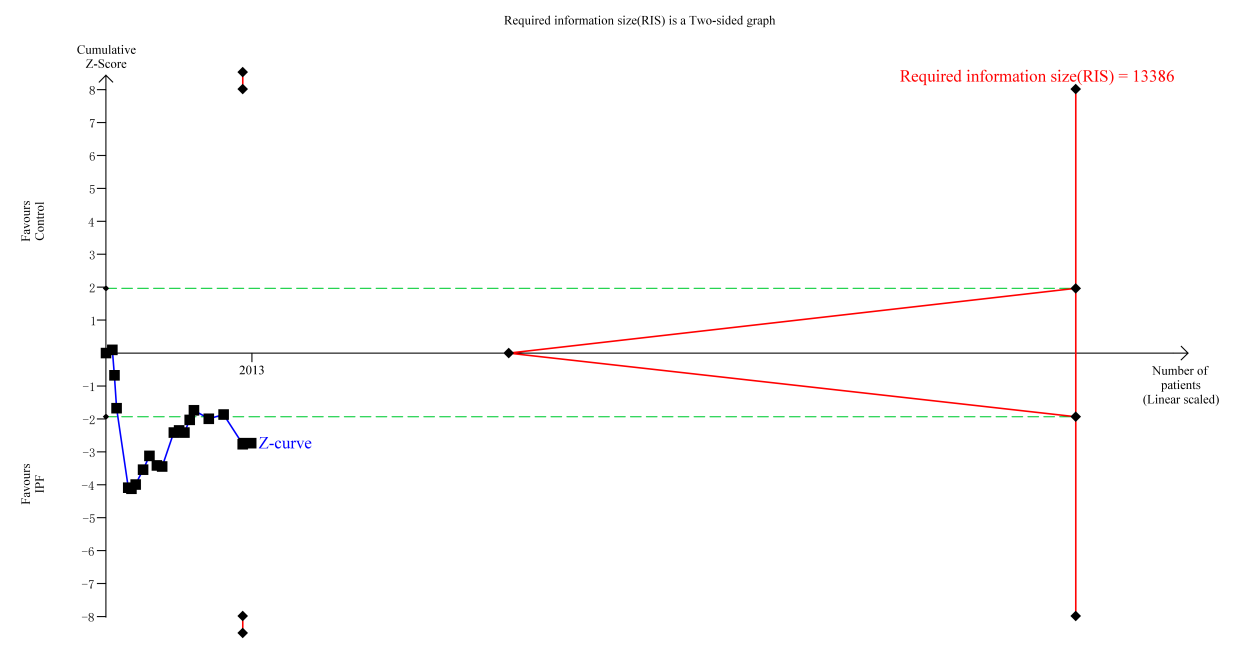
**

**Figure S11 Trial sequential analysis of ACE polymorphism and COPD risk using the additive genetic model (DD vs. II) (Adjusted Boundaries Sketch).**

**Note: The combined sample size(N=2013) did not exceed RIS(N=13386), and the cumulative Z curve crossed the conventional boundary and did not cross the TSA boundary.**

**
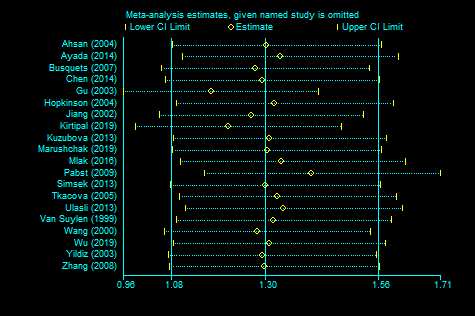
**

**Figure S12 Influence analysis results of DD vs. II**

**
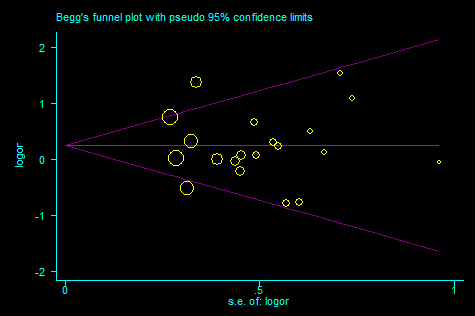
**

**Figure S13 DD vs. II funnel chart generated by Begg's Test**

**
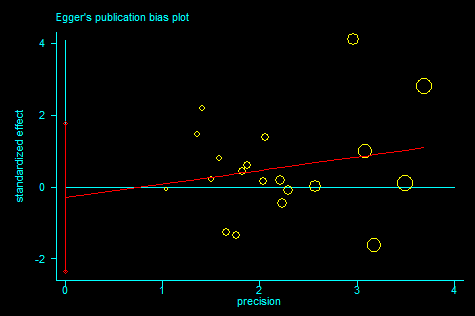
**

**Figure S14 DD vs. II funnel chart of bias generation detected by Egger's test**

**
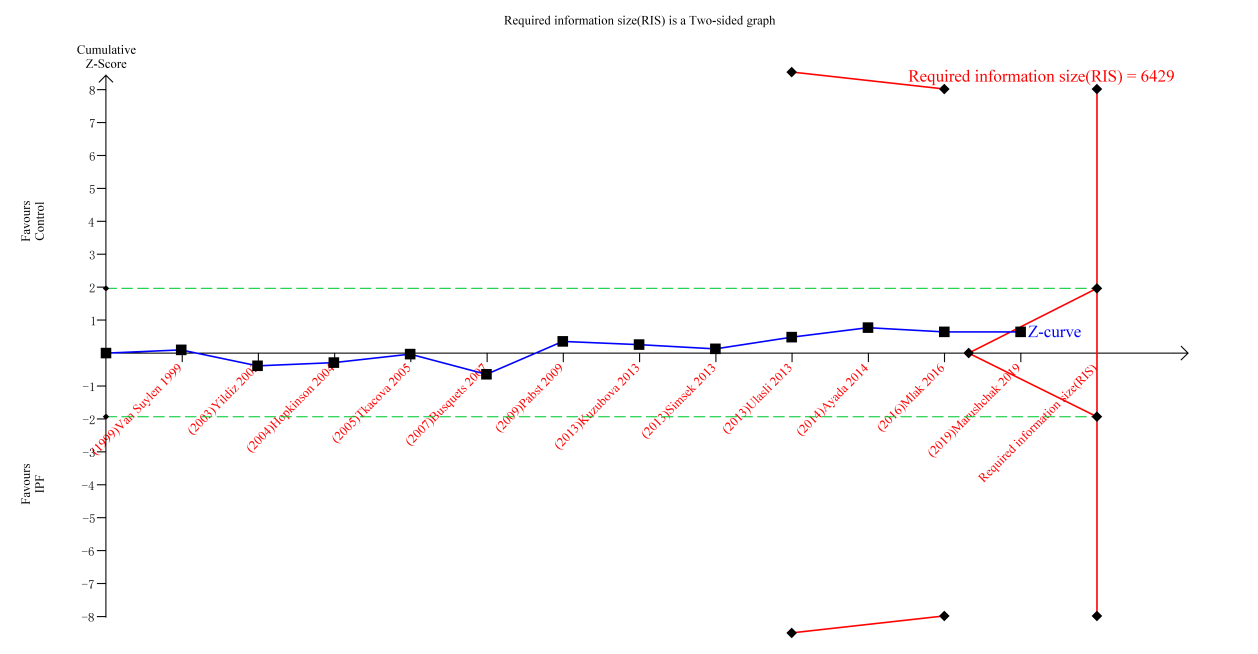
**

**Figure S15 Trial sequential analysis of ACE polymorphism and COPD risk in Caucasian using the additive genetic model (DD vs. II)(Adjusted Boundaries Print)**

**
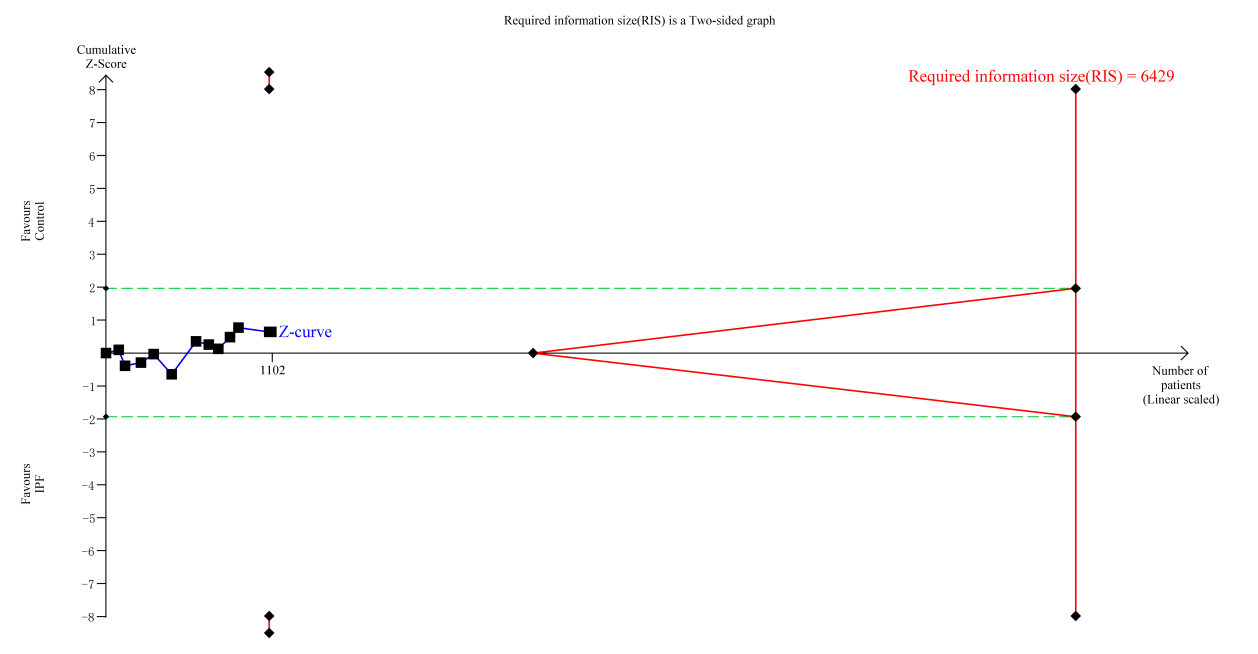
**

**Figure S16 Trial sequential analysis of ACE polymorphism and COPD risk in Caucasian using the additive genetic model (DD vs. II)(Adjusted Boundaries Sketch)**

**
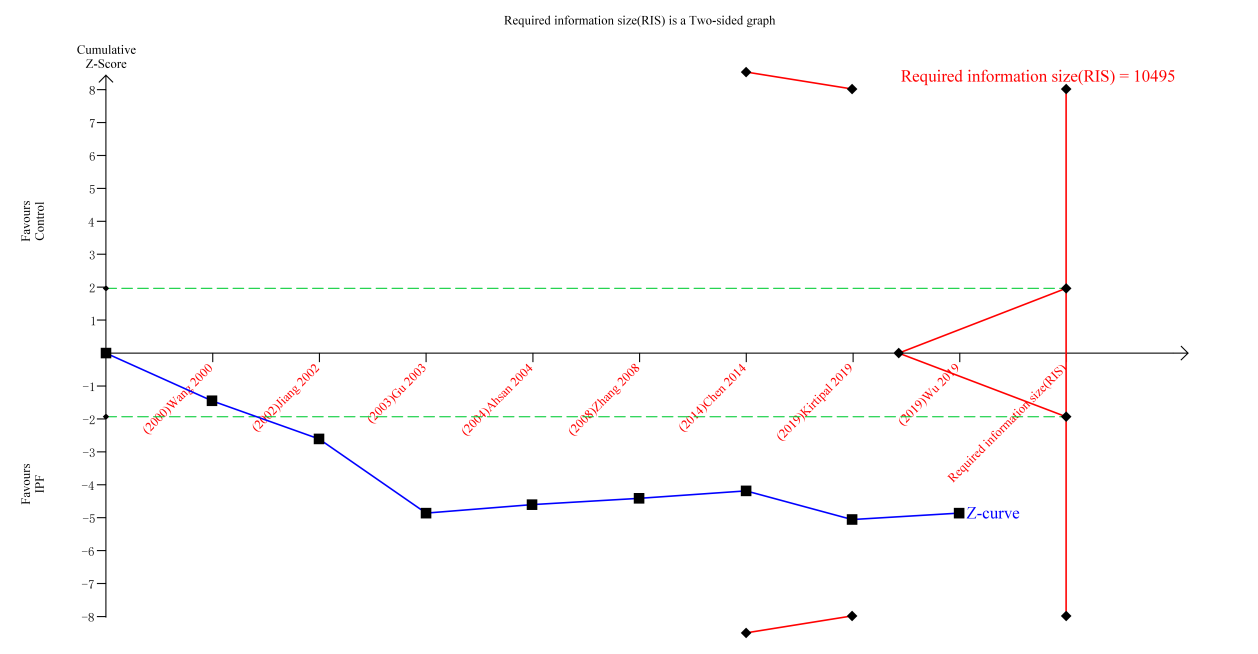
**

**Figure S17 Trial sequential analysis of ACE polymorphism and COPD risk in Asian using the additive genetic model (DD vs. II)(Adjusted Boundaries Print)**

**
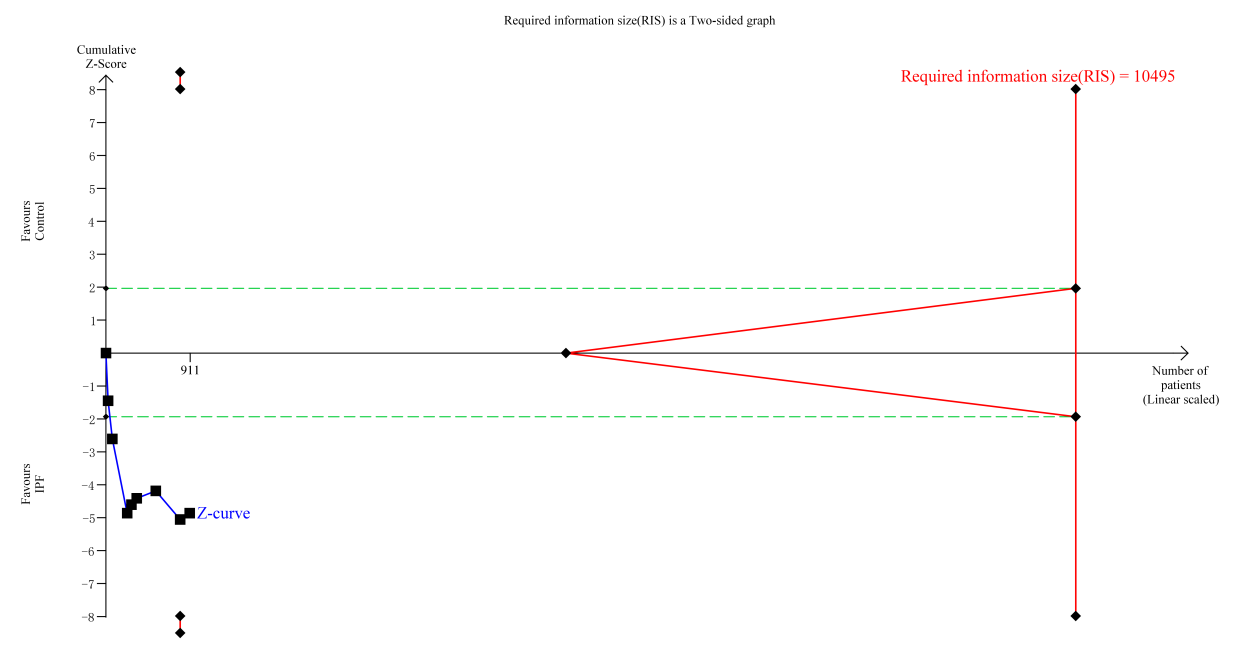
**

**Figure S18 Trial sequential analysis of ACE polymorphism and COPD risk in Asian using the additive genetic model (DD vs. II)(Adjusted Boundaries Sketch)**

**
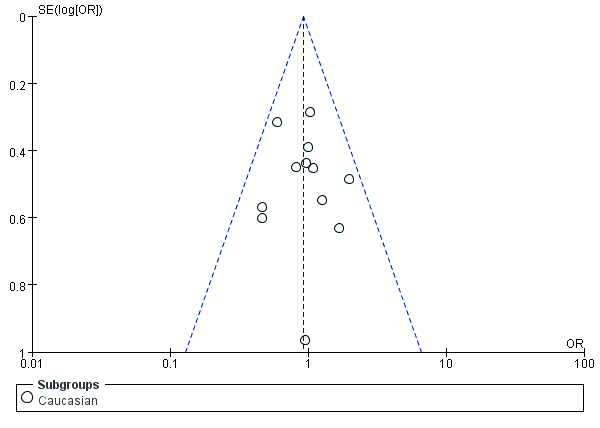
**

**Figure S19 Inverted funnel chart of DD vs. II of Caucasian**

**
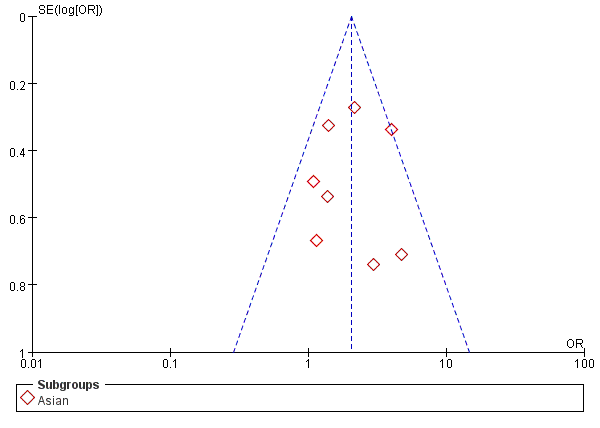
**

**Figure S20 Inverted funnel chart of DD vs. II of Asian**

**ID vs. II**

**
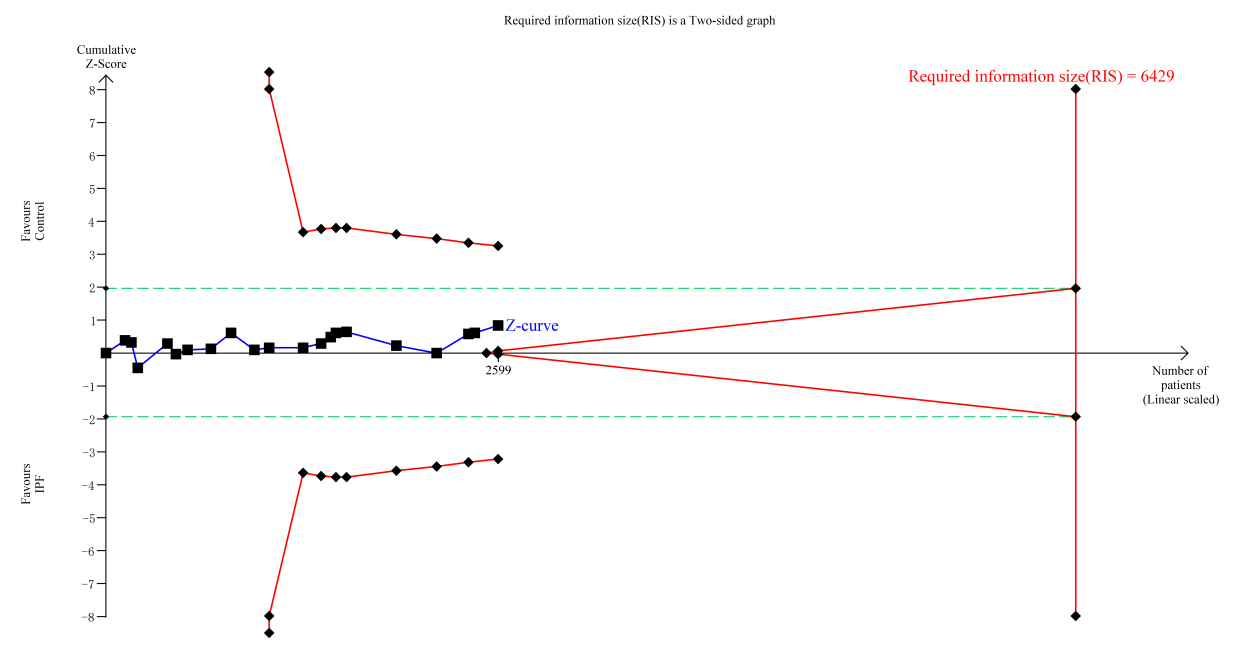
**

**Figure S21 Trial sequential analysis of ACE polymorphism and COPD risk using the heterozygous genetic model(ID vs. II)(Adjusted Boundaries Sketch).**

**Note: The combined sample size(N=2599) did not exceed RIS(N=6429), and the cumulative Z curve did not cross the conventional boundary and the TSA boundary.**

**
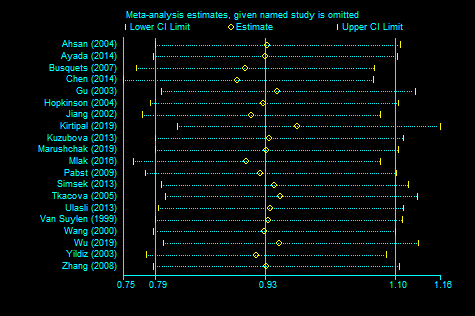
**

**Figure S22 Influence analysis results of ID vs. II**

**
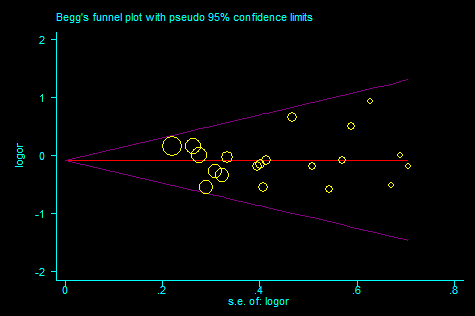
**

**Figure S23 ID vs. II funnel chart generated by Begg's Test**

**
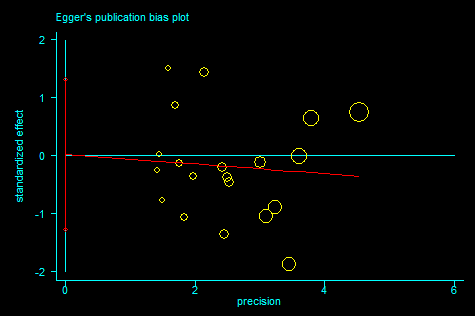
**

**Figure S24 ID vs. II funnel chart of bias generation detected by Egger's test**

**
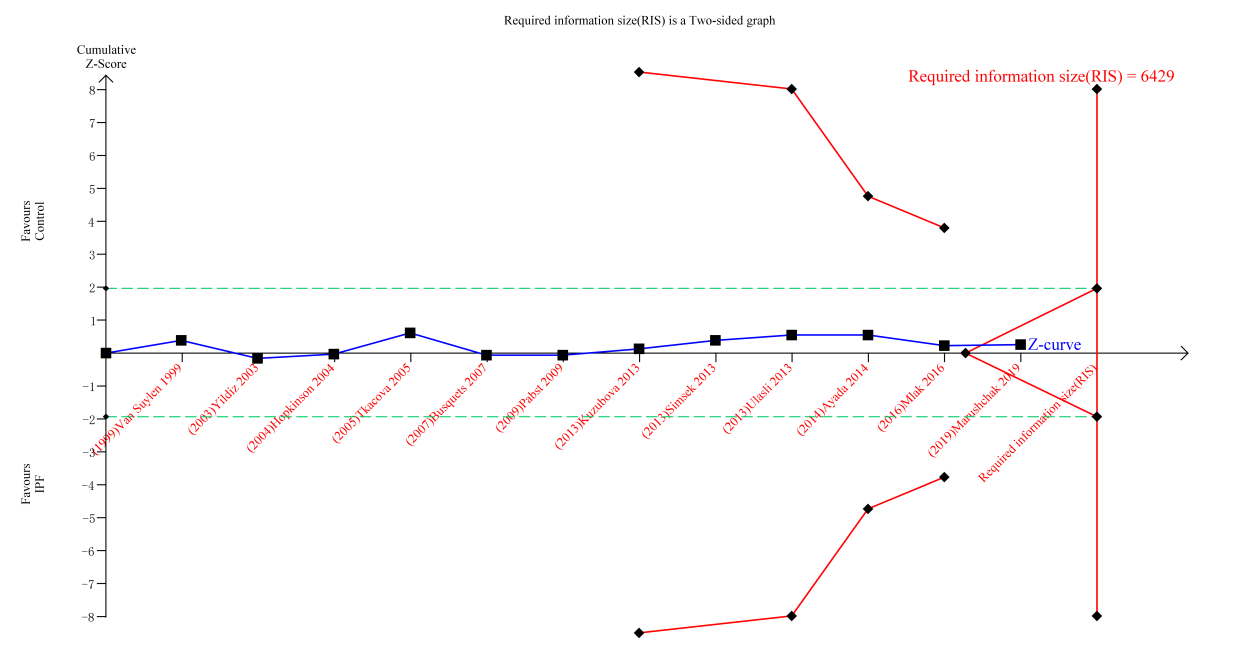
**

**Figure S25 Trial sequential analysis of ACE polymorphism and COPD risk in Caucasian using the heterozygous genetic model (ID vs. II)(Adjusted Boundaries Print)**

**
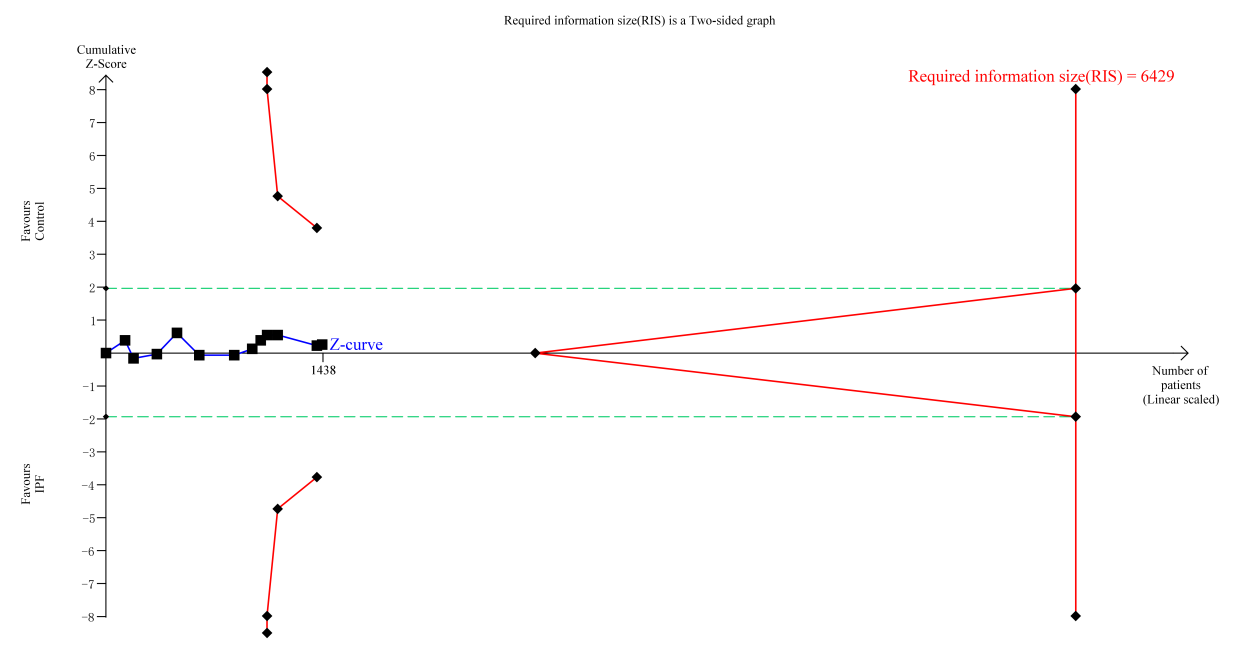
**

**Figure S26 Trial sequential analysis of ACE polymorphism and COPD risk in Caucasian using the heterozygous genetic model (ID vs. II)(Adjusted Boundaries Sketch)**

**
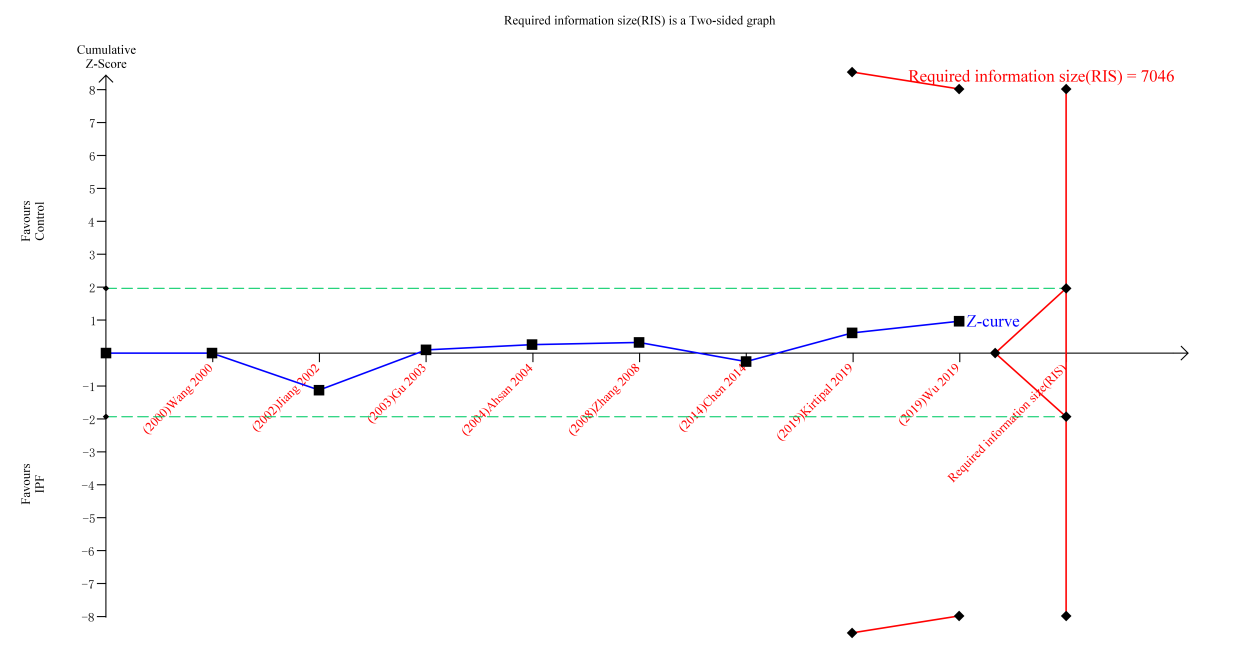
**

**Figure S27 Trial sequential analysis of ACE polymorphism and COPD risk in Asian using the heterozygous genetic model (ID vs. II)(Adjusted Boundaries Print)**

**
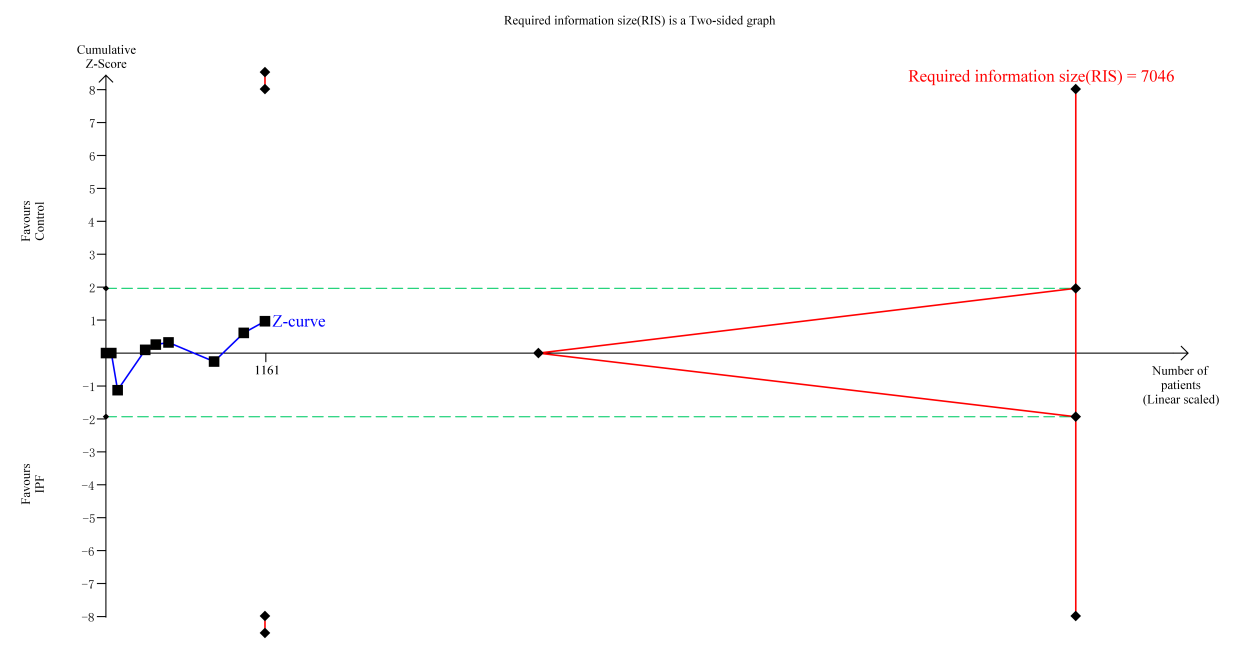
**

**Figure S28 Trial sequential analysis of ACE polymorphism and COPD risk in Asian using the heterozygous genetic model (ID vs. II)(Adjusted Boundaries Sketch)**

**
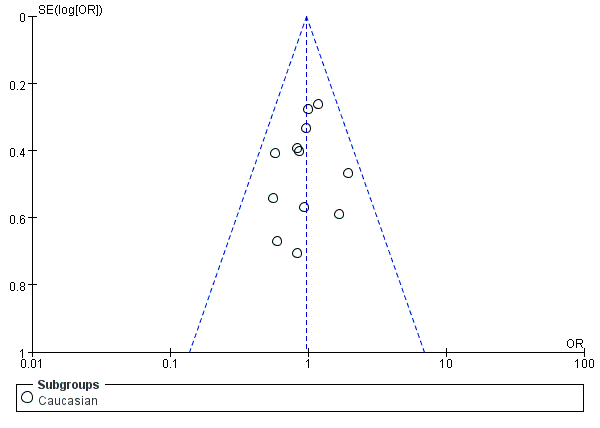
**

**Figure S29 Inverted funnel chart of ID vs. II of Caucasian**

**
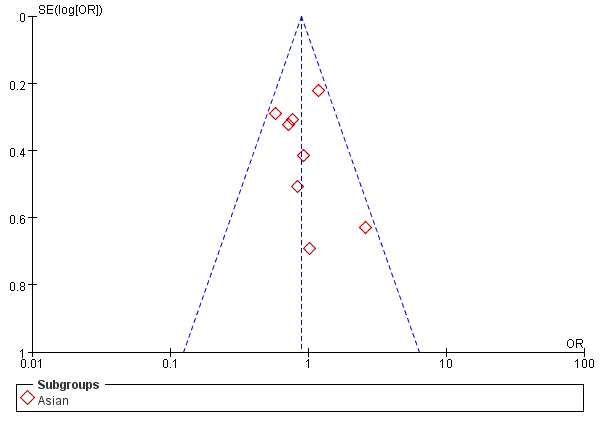
**

**Figure S30 Inverted funnel chart of ID vs. II of Asian**

**DD+ID vs. II**

**
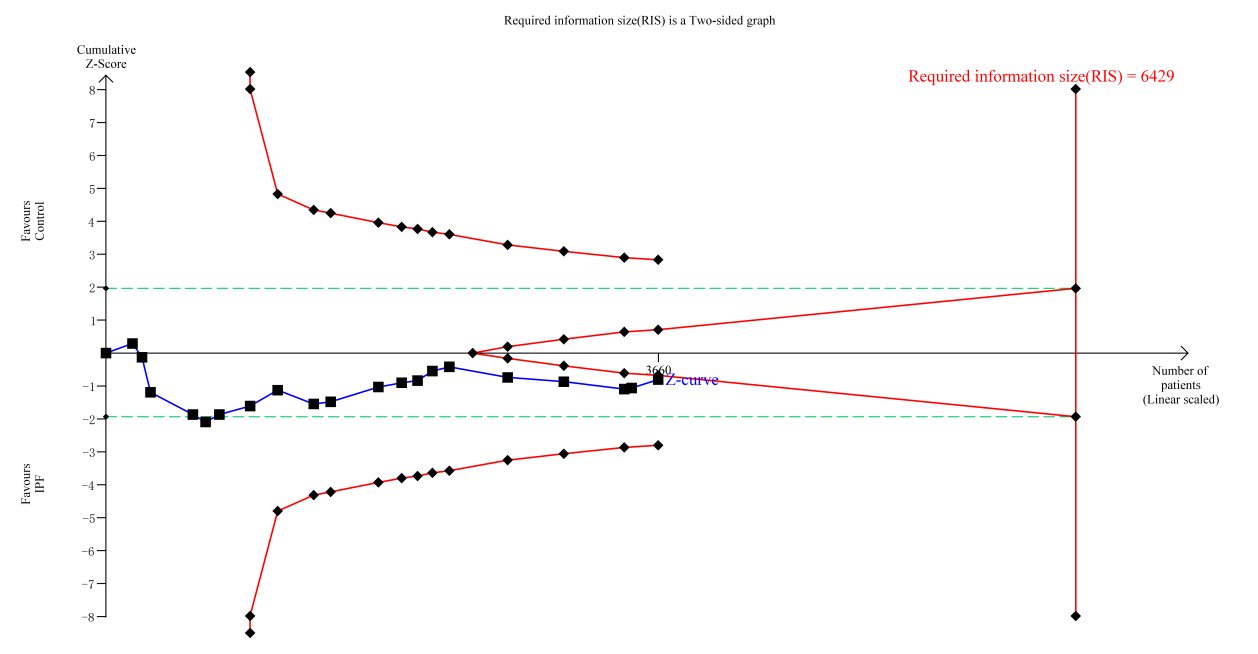
**

**Figure S31 Trial sequential analysis of ACE polymorphism and COPD risk using the dominant genetic model (DD+ID vs. II) (Adjusted Boundaries Sketch).**

**Note: The combined sample size(N=3660) did not exceed RIS(N=6429), and the cumulative Z curve crossed the conventional boundary and did not cross the TSA boundary.**

**
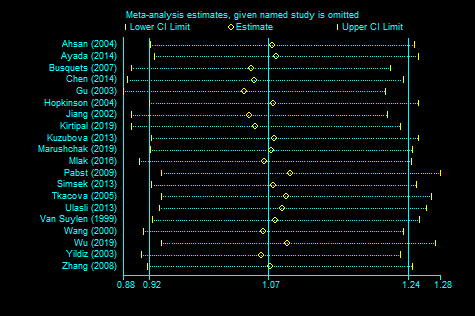
**

**Figure S32 Influence analysis results of DD+ID vs. II**

**
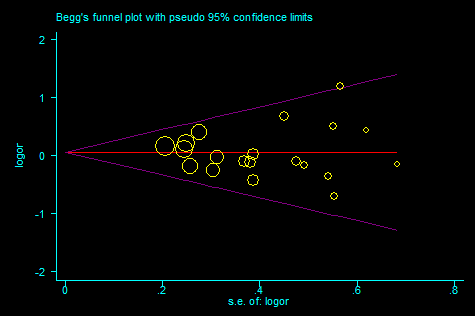
**

**Figure S33 DD+ID vs. II funnel chart generated by Begg's Test**

**
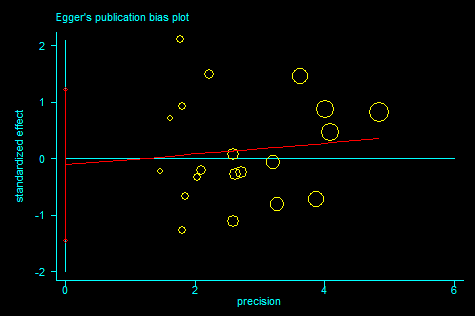
**

**Figure S34 DD+ID vs. II funnel chart of bias generation detected by Egger's test**

**
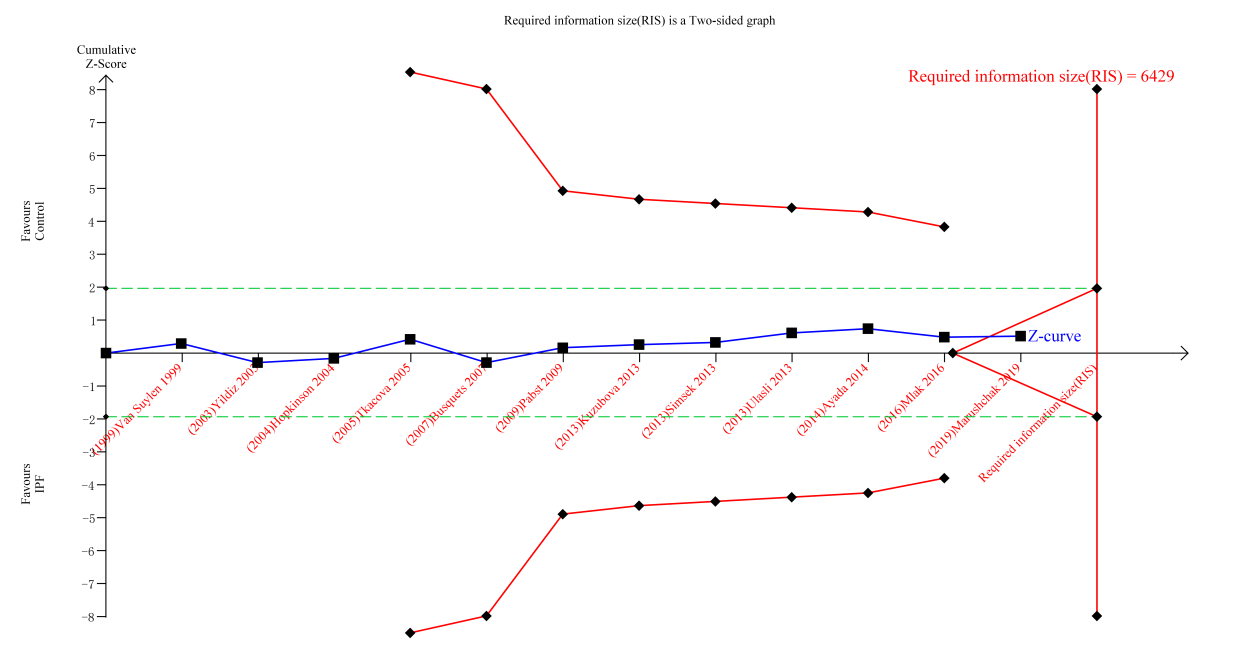
**

**Figure S35 Trial sequential analysis of ACE polymorphism and COPD risk in Caucasian using the dominant genetic model (DD+ID vs. II)(Adjusted Boundaries Print)**

**
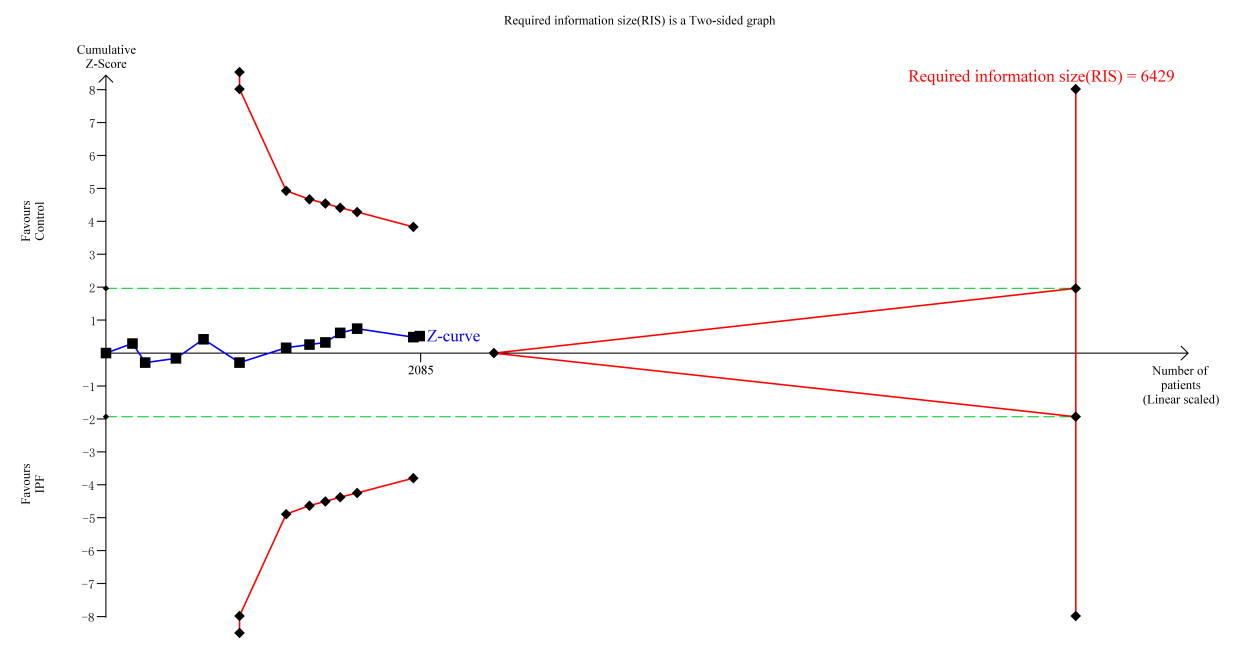
**

**Figure S36 Trial sequential analysis of ACE polymorphism and COPD risk in Caucasian using the dominant genetic model (DD+ID vs. II)(Adjusted Boundaries Sketch)**

**
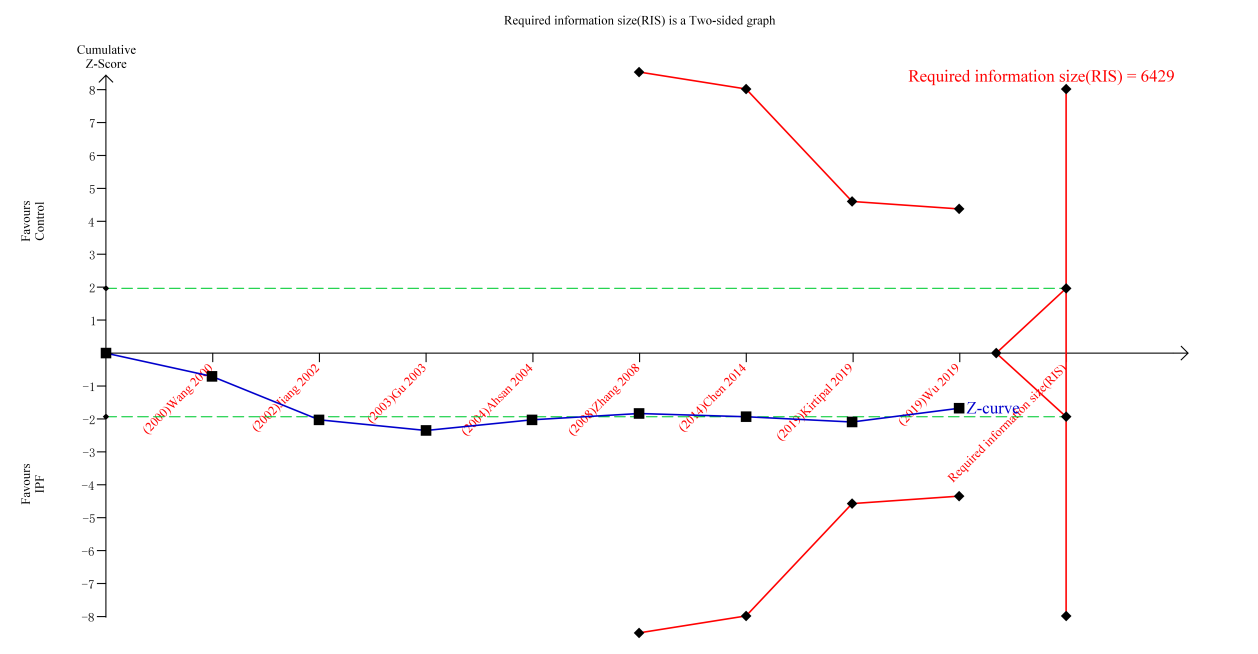
**

**Figure S37 Trial sequential analysis of ACE polymorphism and COPD risk in Asian using the dominant genetic model (DD+ID vs. II)(Adjusted Boundaries Print)**

**
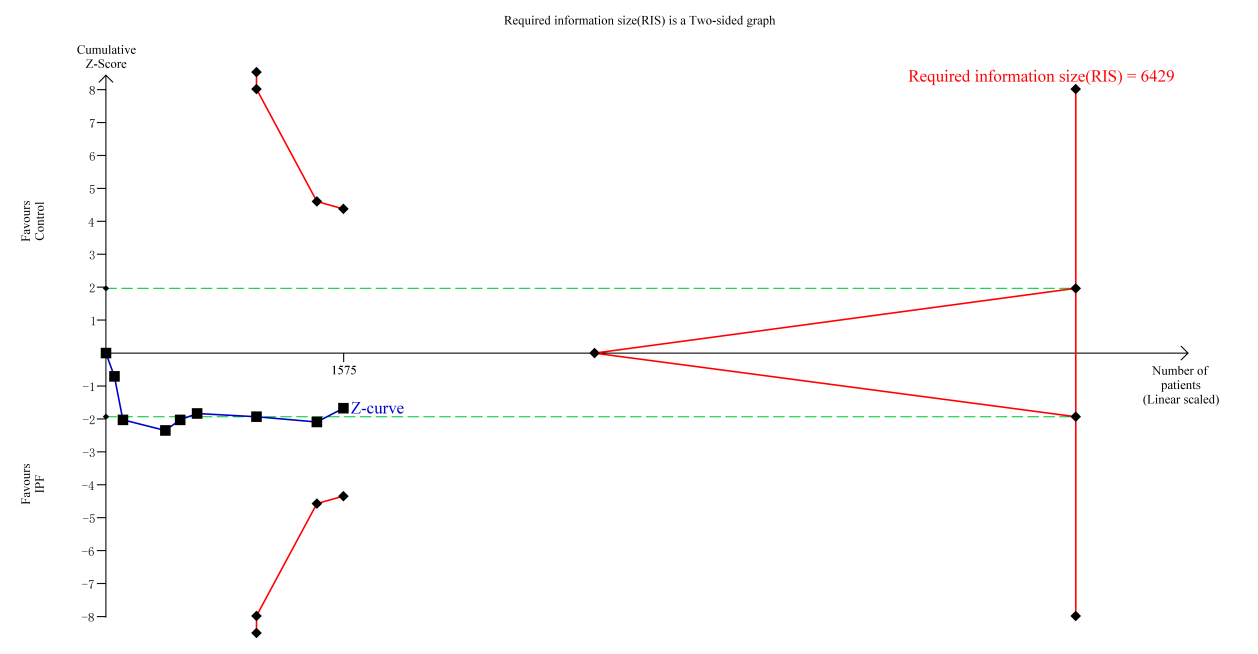
**

**Figure S38 Trial sequential analysis of ACE polymorphism and COPD risk in Asian using the dominant genetic model (DD+ID vs. II)(Adjusted Boundaries Sketch)**

**
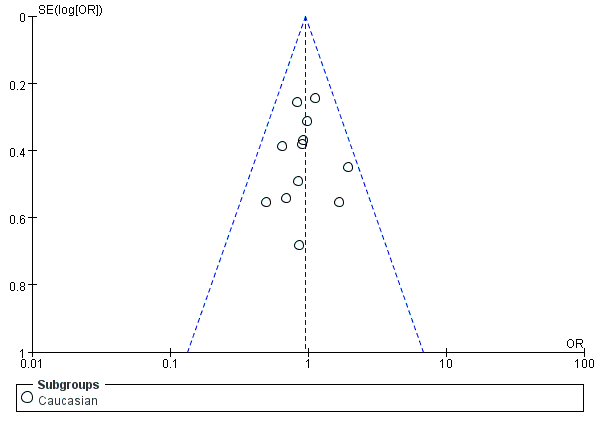
**

**Figure S39 Inverted funnel chart of DD+ID vs. II of Caucasian**

**
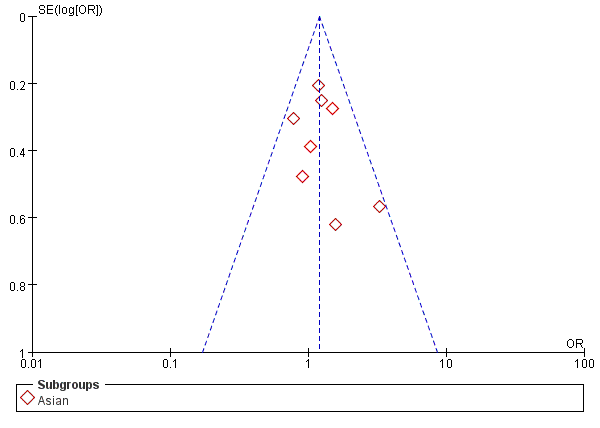
**

**Figure S40 Inverted funnel chart of DD+ID vs. II of Asian**

**DD vs. II+ID**

**
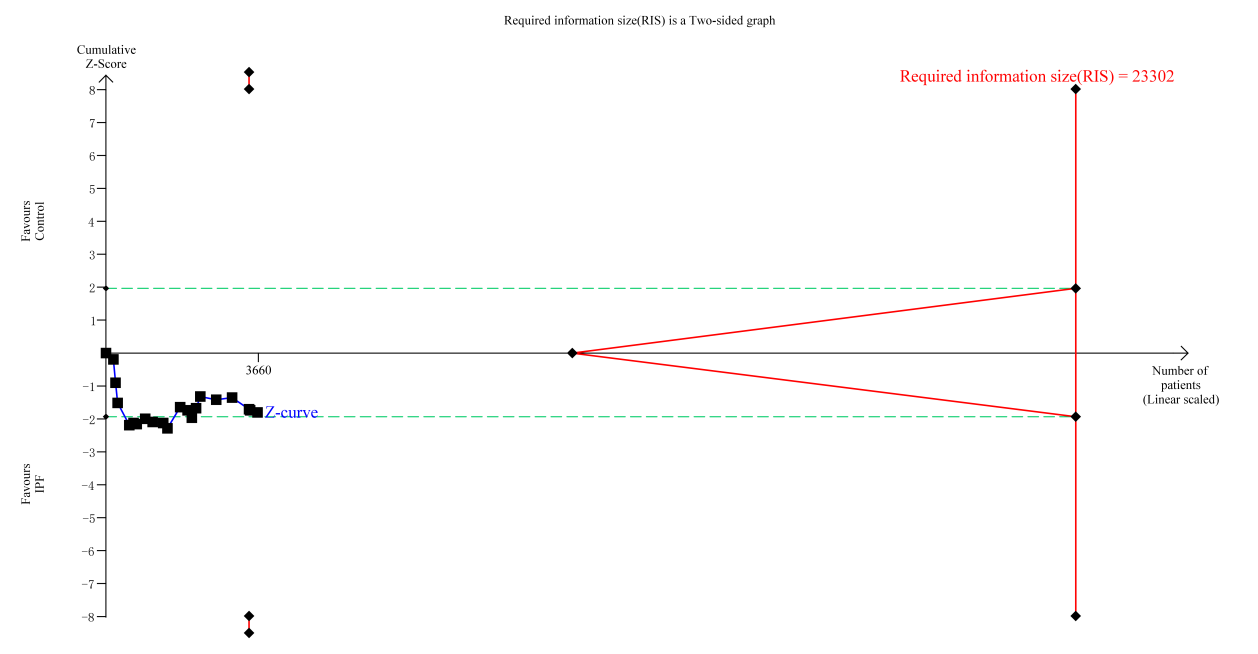
**

**Figure S41 Trial sequential analysis of ACE polymorphism and COPD risk using the recessive genetic model (DD vs. II+ID)(Adjusted Boundaries Sketch).**

**Note: The combined sample size(N=3660) exceeded RIS(N=23302),and the cumulative Z curve crossed the conventional boundary and did not cross the TSA boundary.**

**
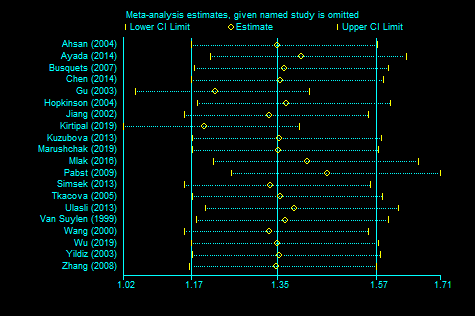
**

**Figure S42 Influence analysis results of DD vs. II+ID**

**
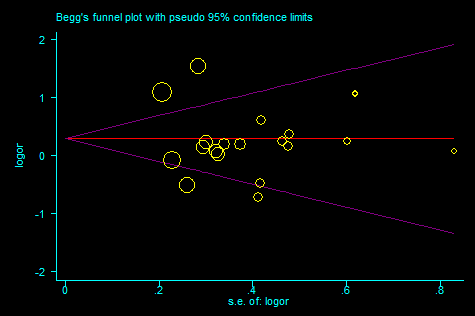
**

**Figure S43 DD vs. II+ID funnel chart generated by Begg's Test**

**
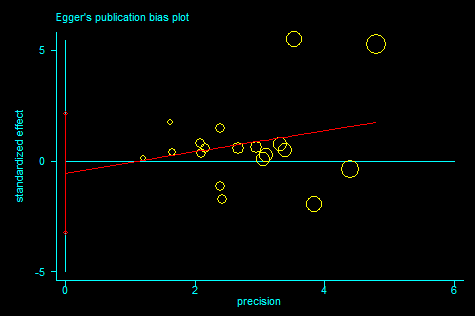
**

**Figure S44 DD vs. II+ID funnel chart of bias generation detected by Egger's test**

**
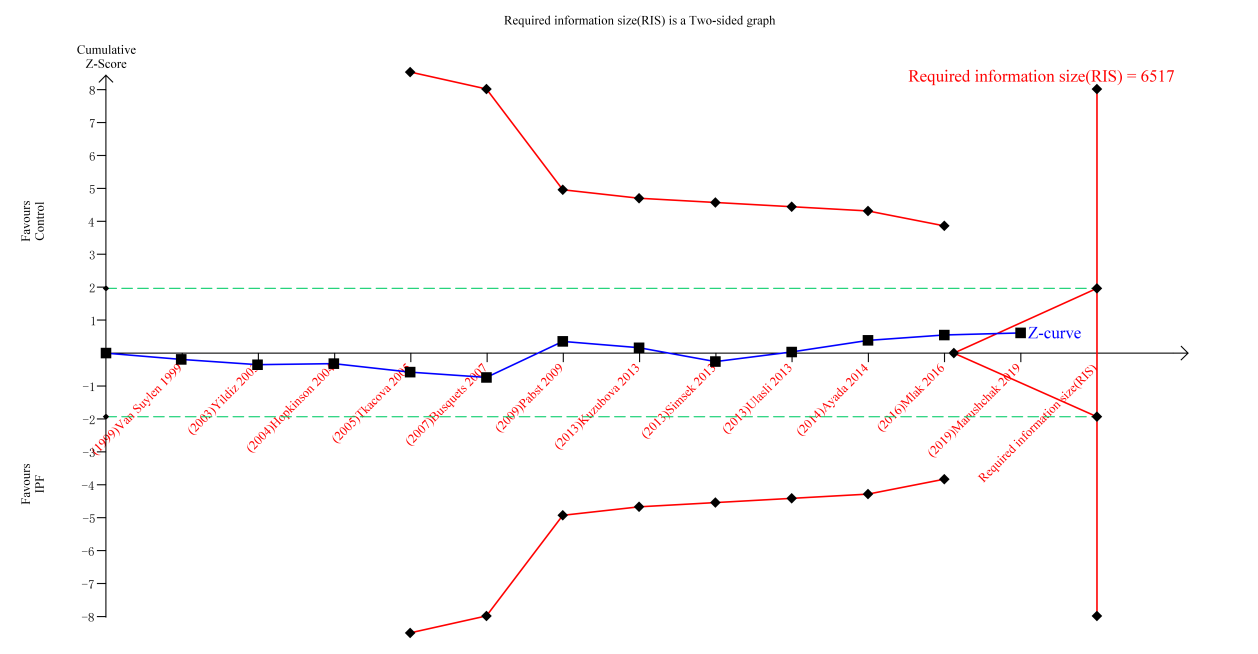
**

**Figure S45 Trial sequential analysis of ACE polymorphism and COPD risk in Caucasian using the recessive genetic model (DD vs. II+ID)(Adjusted Boundaries Print)**

**
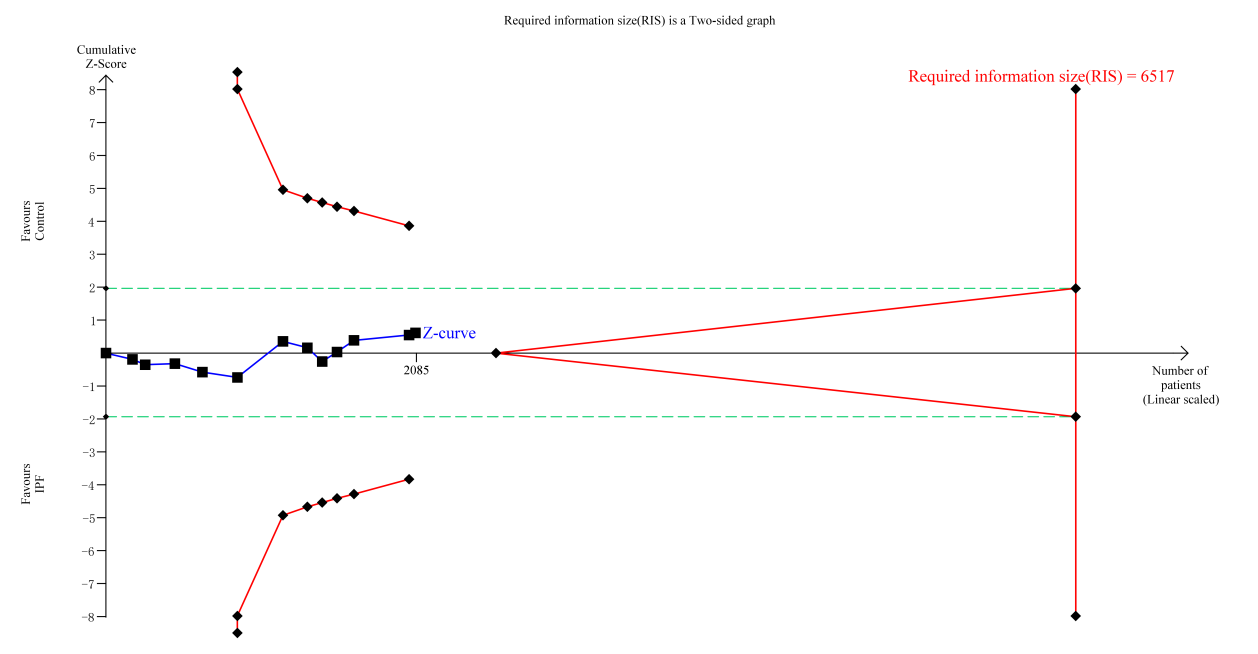
**

**Figure S46 Trial sequential analysis of ACE polymorphism and COPD risk in Caucasian using the recessive genetic model (DD vs. II+ID)(Adjusted Boundaries Sketch)**

**
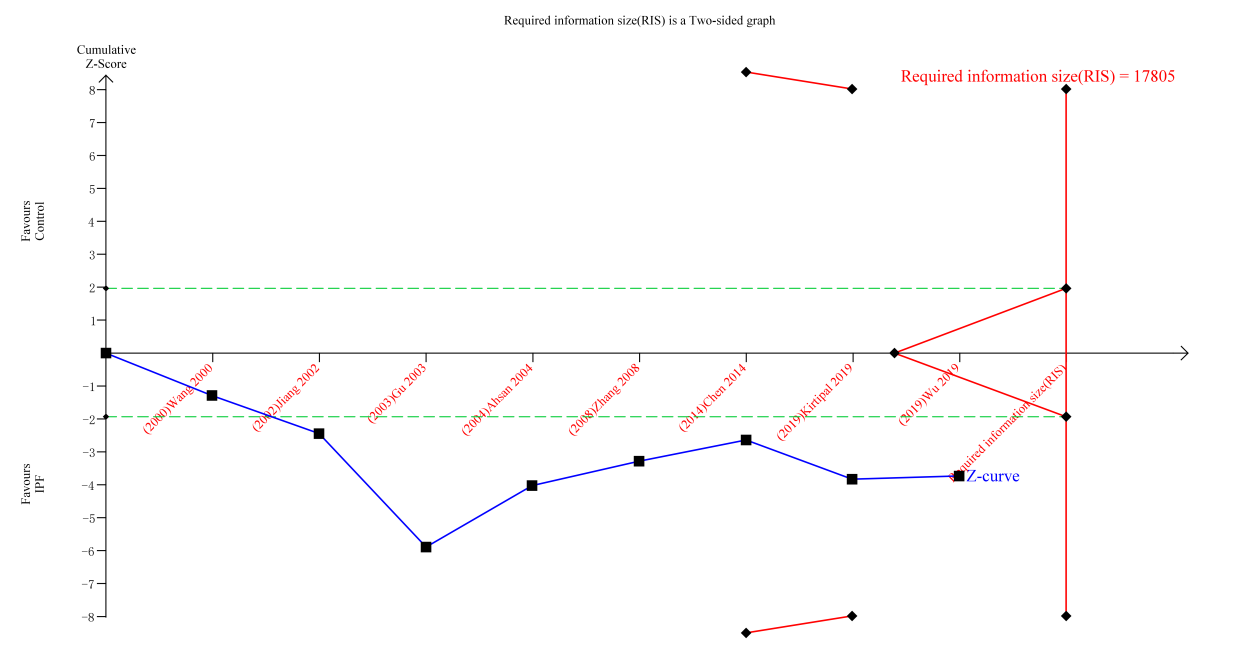
**

**Figure S47 Trial sequential analysis of ACE polymorphism and COPD risk in Asian using the recessive genetic model (DD vs. II+ID)(Adjusted Boundaries Print)**

**
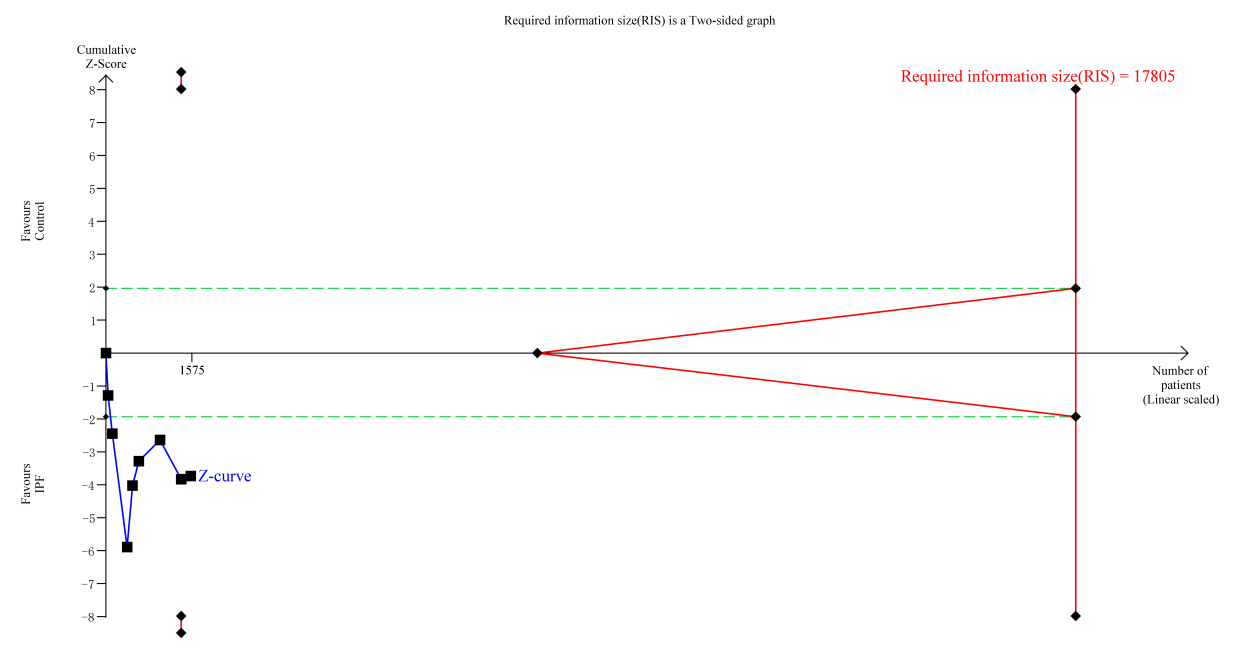
**

**Figure S48 Trial sequential analysis of ACE polymorphism and COPD risk in Asian using the recessive genetic model (DD vs. II+ID)(Adjusted Boundaries Sketch)**

**
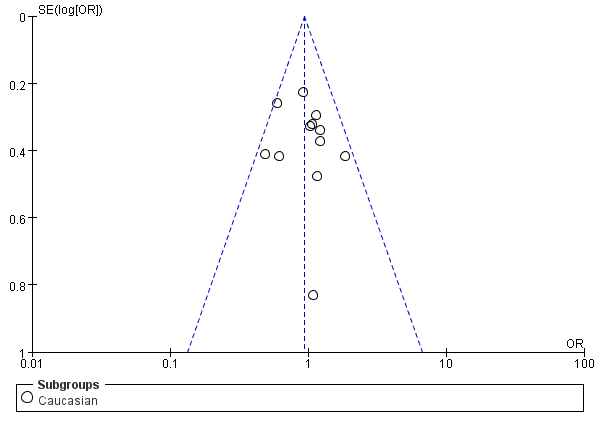
**

**Figure S49 Inverted funnel chart of DD vs. II+ID of Caucasian**

**
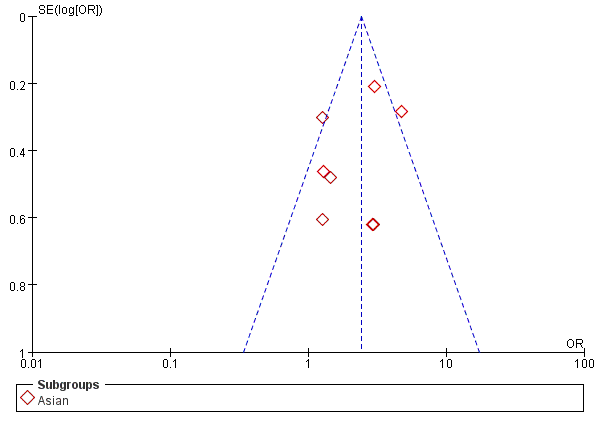
**

**Figure S50 Inverted funnel chart of DD vs. II+ID of Asian**

**Table S1 PubMed search strategy**

| Number | Search Terms |
| --- | --- |
| #1 | Mesh descriptor: (Chronic obstructive pulmonary disease) explode all trees |
| #2 | (COPD[Title/Abstract]) OR (Chronic obstructive pulmonary disease[Title/Abstract]) |
| #3 | Or 1-2 |
| #4 | Mesh descriptor: (Angiotensin converting enzyme) explode all trees |
| #5 | (ACE[Title/Abstract]) OR Angiotensin converting enzyme[Title/Abstract]) |
| #6 | Or 4-5 |
| #7 | Mesh descriptor: (polymorphism) explode all trees |
| #8 | 3 and 6 and 7 |

**Table S2 Basic features of the included study (2)**

| Studies | Genotyping methods | COPD | | | | | Controls | | | | | Hardy-Weinberg | |
| --- | --- | --- | --- | --- | --- | --- | --- | --- | --- | --- | --- | --- | --- |
|  |  | II | ID | DD | I | D | II | ID | DD | I | D | χ^2^ | PHWE |
| Ahsan[11] | PCR | 10 | 12 | 5 | 32 | 22 | 23 | 33 | 10 | 79 | 53 | 0.033 | 0.856 |
| Ayada[18] | PCR | 8 | 26 | 13 | 42 | 52 | 8 | 28 | 28 | 44 | 84 | 0.076 | 0.783 |
| Busquets[19] | PCR | 7 | 40 | 27 | 54 | 94 | 27 | 79 | 53 | 133 | 185 | 0.022 | 0.882 |
| Chen[20] | PCR | 72 | 90 | 28 | 234 | 146 | 82 | 87 | 23 | 251 | 133 | 0.011 | 0.918 |
| Gu[21] | PCR | 28 | 37 | 57 | 93 | 151 | 49 | 85 | 25 | 183 | 135 | 0.231 | 0.631 |
| Hopkinson[10] | PCR | 29 | 49 | 25 | 107 | 99 | 28 | 49 | 24 | 105 | 97 | 0.025 | 0.874 |
| Jiang[22] | PCR | 7 | 12 | 11 | 26 | 34 | 15 | 10 | 5 | 40 | 20 | 0.268 | 0.605 |
| Kirtipal[23] | PCR | 37 | 44 | 119 | 118 | 282 | 44 | 90 | 66 | 178 | 222 | 0.241 | 0.623 |
| Kuzubova[24] | PCR | 17 | 29 | 17 | 63 | 63 | 24 | 49 | 22 | 97 | 93 | 0.028 | 0.868 |
| Marushchak[25] | PCR | 7 | 14 | 4 | 28 | 22 | 5 | 12 | 3 | 22 | 18 | 0.125 | 0.723 |
| Mlak[26] | PCR | 47 | 99 | 60 | 193 | 219 | 41 | 73 | 51 | 155 | 175 | 0.423 | 0.515 |
| Pabst[27] | PCR | 43 | 76 | 33 | 162 | 142 | 39 | 69 | 50 | 147 | 169 | 0.445 | 0.505 |
| Simsek[5] | PCR | 15 | 20 | 31 | 50 | 82 | 8 | 19 | 13 | 35 | 45 | 0.082 | 0.775 |
| Tkacova[12] | PCR | 15 | 31 | 20 | 61 | 71 | 19 | 68 | 31 | 106 | 130 | 0.711 | 0.399 |
| Ulasli[28] | PCR | 11 | 11 | 28 | 33 | 67 | 6 | 10 | 33 | 22 | 76 | 2.178 | 0.14 |
| Van Suylen[29] | PCR | 17 | 43 | 27 | 77 | 97 | 17 | 50 | 28 | 84 | 106 | 0.137 | 0.711 |
| Wang[30] | PCR | 5 | 7 | 8 | 17 | 23 | 13 | 18 | 7 | 44 | 32 | 0.06 | 0.807 |
| Wu[31] | PCR | 44 | 38 | 13 | 126 | 64 | 33 | 40 | 9 | 106 | 58 | 0.025 | 0.874 |
| Yildiz[32] | PCR | 7 | 21 | 14 | 35 | 49 | 10 | 18 | 12 | 38 | 42 | 0.069 | 0.793 |
| Zhang[33] | PCR | 21 | 27 | 13 | 69 | 53 | 20 | 28 | 9 | 68 | 46 | 0.038 | 0.845 |

Note: PCR: polymerase chain reaction; PHWE: P value of Hardy-Weinberg equilibrium; COPD: Chronic obstructive pulmonary disease.

**Table S3 Newcastle Ottawa scale (NOS)**

| **Studies** | **Select** | | | | **Comparability^a^** | **Expose** | | | **Total score^b^** |
| --- | --- | --- | --- | --- | --- | --- | --- | --- | --- |
|  | **1** | **2** | **3** | **4** | **5** | **6** | **7** | **8** |  |
|  | **Ⅰ** | **Ⅱ** | **Ⅲ** | **Ⅳ** | **Ⅴ** | **Ⅵ** | **Ⅶ** | **Ⅷ** |  |
| Ahsan[11] | ☆ | ☆ | ☆ | ☆ | ☆☆ | ☆ |  |  | 7☆ |
| Ayada[18] | ☆ | ☆ | ☆ | ☆ | ☆☆ | ☆ |  |  | 7☆ |
| Busquets[19] | ☆ | ☆ | ☆ | ☆ | ☆☆ | ☆ |  |  | 7☆ |
| Chen[20] | ☆ | ☆ | ☆ | ☆ | ☆☆ | ☆ | ☆ |  | 8☆ |
| Gu[21] | ☆ | ☆ | ☆ | ☆ | ☆☆ | ☆ | ☆ |  | 8☆ |
| Hopkinson[10] | ☆ | ☆ | ☆ | ☆ | ☆☆ | ☆ | ☆ |  | 8☆ |
| Jiang[22] | ☆ | ☆ | ☆ | ☆ | ☆☆ | ☆ | ☆ |  | 8☆ |
| Kirtipal[23] | ☆ | ☆ | ☆ | ☆ | ☆☆ | ☆ |  |  | 7☆ |
| Kuzubova[24] | ☆ | ☆ | ☆ | ☆ | ☆☆ | ☆ | ☆ |  | 8☆ |
| Marushchak[25] | ☆ | ☆ | ☆ | ☆ | ☆☆ | ☆ |  |  | 7☆ |
| Mlak[26] | ☆ | ☆ | ☆ | ☆ | ☆☆ | ☆ | ☆ |  | 8☆ |
| Pabst[27] | ☆ | ☆ | ☆ | ☆ | ☆☆ | ☆ | ☆ |  | 8☆ |
| Simsek[5] | ☆ | ☆ | ☆ | ☆ | ☆☆ | ☆ | ☆ |  | 8☆ |
| Tkacova[12] | ☆ | ☆ | ☆ | ☆ | ☆☆ | ☆ | ☆ |  | 8☆ |
| Ulasli[28] | ☆ | ☆ | ☆ | ☆ | ☆ | ☆ | ☆ |  | 7☆ |
| Van Suylen[29] | ☆ | ☆ | ☆ | ☆ | ☆☆ | ☆ | ☆ |  | 8☆ |
| Wang[30] | ☆ | ☆ | ☆ | ☆ | ☆☆ | ☆ | ☆ |  | 8☆ |
| Wu[31] | ☆ | ☆ | ☆ | ☆ | ☆☆ | ☆ | ☆ |  | 8☆ |
| Yildiz[32] | ☆ | ☆ | ☆ | ☆ | ☆☆ | ☆ | ☆ |  | 8☆ |
| Zhang[33] | ☆ | ☆ | ☆ | ☆ | ☆☆ | ☆ | ☆ |  | 8☆ |

Note: a: Two stars with the highest comparability;b: Full score is 9☆.1-8:Case-control studies (CC);Ⅰ-Ⅷ:Cohort studies(CS).

1. Case definition;2: Case manifestations;3. Selection of control group;4. Definition of control group;5: Choose the most important/second most important factor;6. Determination of exposure;7. Methods for determining cases and control groups;

8: No response rate.

I: representativeness of exposure; II: selection of non exposed persons; III: Determination of exposure; IV: proof of no interesting results at the beginning; V: comparability; VI: evaluation of results; VII: long enough follow-up time; VIII: adequacy of follow-up.

**Table S4 Influence analysis results of D vs.I**

| Study omitted | Estimate | 95% Conf. Interval | |
| --- | --- | --- | --- |
| Ahsan (2004) | 1.1647792 | 1.0588499 | 1.2813058 |
| Ayada (2014) | 1.1827744 | 1.0747145 | 1.3016994 |
| Busquets (2007) | 1.1565171 | 1.0495701 | 1.2743617 |
| Chen (2014) | 1.1597956 | 1.0499027 | 1.2811909 |
| Gu (2003) | 1.1002517 | 0.99716699 | 1.213993 |
| Hopkinson (2004) | 1.1724396 | 1.0638051 | 1.2921678 |
| Jiang (2002) | 1.1457068 | 1.041714 | 1.2600809 |
| Kirtipal (2019) | 1.0944717 | 0.99043226 | 1.2094399 |
| Kuzubova (2013) | 1.1673452 | 1.0600058 | 1.2855541 |
| Marushchak (2019) | 1.1644459 | 1.0589976 | 1.2803941 |
| Mlak (2016) | 1.1816497 | 1.0694629 | 1.3056051 |
| Pabst (2009) | 1.2110085 | 1.096911 | 1.3369743 |
| Simsek (2013) | 1.1584949 | 1.0528119 | 1.2747866 |
| Tkacova (2005) | 1.1736689 | 1.0654851 | 1.292837 |
| Ulasli (2013) | 1.1803198 | 1.0728525 | 1.298552 |
| Van Suylen (1999) | 1.1712707 | 1.0631329 | 1.290408 |
| Wang (2000) | 1.1533233 | 1.0487586 | 1.2683134 |
| Wu (2019) | 1.1742613 | 1.0661577 | 1.2933263 |
| Yildiz (2003) | 1.159191 | 1.0536762 | 1.2752719 |
| Zhang (2008) | 1.162501 | 1.0561755 | 1.2795303 |
| Combined | 1.1615942 | 1.0570463 | 1.2764825 |

**Table S5 Results of Begg's Test and Egger's Test to detect D vs.I bias in different populations**

| Ethnicity | n | Begg's | | Begg's | | cont. corr. | | Egger's | |
| --- | --- | --- | --- | --- | --- | --- | --- | --- | --- |
|  |  | score | s.d. | z | p | z | p | bias | p |
| Asian | 8 | 0 | 8.083 | 0.00 | 1.000 | -0.12 | 1.000 | -0.37 | 0.851 |
| Caucasian | 12 | -6 | 14.583 | -0.41 | 0.681 | 0.34 | 0.732 | -0.02 | 0.982 |
| overall | 20 | -6 | 16.673 | -0.36 | 0.719 | -0.30 | 0.764 | -0.11 | 0.907 |

**Table S6 Influence analysis results of DD vs. II**

| Study omitted | Estimate | 95% Conf. Interval | |
| --- | --- | --- | --- |
| Ahsan (2004) | 1.3002063 | 1.0775454 | 1.5688772 |
| Ayada (2014) | 1.3319124 | 1.1031092 | 1.6081731 |
| Busquets (2007) | 1.2739784 | 1.0537276 | 1.5402662 |
| Chen (2014) | 1.2889351 | 1.0612186 | 1.565515 |
| Gu (2003) | 1.1698828 | 0.96244091 | 1.4220361 |
| Hopkinson (2004) | 1.317994 | 1.0880485 | 1.5965356 |
| Jiang (2002) | 1.2644693 | 1.0477895 | 1.5259578 |
| Kirtipal (2019) | 1.2091134 | 0.99113613 | 1.4750296 |
| Kuzubova (2013) | 1.3073632 | 1.0809758 | 1.5811626 |
| Marushchak (2019) | 1.3009468 | 1.0792583 | 1.568172 |
| Mlak (2016) | 1.3350862 | 1.0961879 | 1.6260489 |
| Pabst (2009) | 1.4045224 | 1.1547639 | 1.7083001 |
| Simsek (2013) | 1.2977766 | 1.0745521 | 1.5673733 |
| Tkacova (2005) | 1.3254712 | 1.0957874 | 1.6032982 |
| Ulasli (2013) | 1.3387398 | 1.1081551 | 1.6173043 |
| Van Suylen (1999) | 1.3162773 | 1.0879453 | 1.5925303 |
| Wang (2000) | 1.2786198 | 1.0599229 | 1.542441 |
| Wu (2019) | 1.3061268 | 1.0806983 | 1.5785787 |
| Yildiz (2003) | 1.2894577 | 1.0684003 | 1.5562531 |
| Zhang (2008) | 1.2945096 | 1.0716805 | 1.5636705 |
| Combined | 1.2970023 | 1.0769722 | 1.5619857 |

**Table S7 Results of Begg's Test and Egger's Test to detect DD vs. II bias in different populations**

| Ethnicity | n | Begg's | | Begg's | | cont. corr. | | Egger's | |
| --- | --- | --- | --- | --- | --- | --- | --- | --- | --- |
|  |  | score | s.d. | z | p | z | p | bias | p |
| Asian | 8 | 4 | 8.083 | 0.49 | 0.621 | 0.37 | 0.711 | -0.21 | 0.879 |
| Caucasian | 12 | 4 | 14.583 | 0.27 | 0.784 | 0.21 | 0.837 | 0.30 | 0.756 |
| overall | 20 | 8 | 16.673 | 0.48 | 0.631 | 0.42 | 0.675 | 0.13 | 0.865 |

**Table S8 Influence analysis results of ID vs. II**

| Study omitted | Estimate | 95% Conf. Interval | |
| --- | --- | --- | --- |
| Ahsan (2004) | 0.93520564 | 0.79173076 | 1.1046805 |
| Ayada (2014) | 0.93245155 | 0.78977871 | 1.1008981 |
| Busquets (2007) | 0.90669924 | 0.76687485 | 1.0720178 |
| Chen (2014) | 0.89614207 | 0.75029343 | 1.0703421 |
| Gu (2003) | 0.94743979 | 0.79876488 | 1.1237878 |
| Hopkinson (2004) | 0.93016762 | 0.78497738 | 1.1022123 |
| Jiang (2002) | 0.91447502 | 0.77465719 | 1.0795287 |
| Kirtipal (2019) | 0.97360003 | 0.81990248 | 1.1561095 |
| Kuzubova (2013) | 0.93719751 | 0.79218781 | 1.1087512 |
| Marushchak (2019) | 0.9338758 | 0.79148406 | 1.1018845 |
| Mlak (2016) | 0.90770626 | 0.76331705 | 1.0794082 |
| Pabst (2009) | 0.92588151 | 0.77930337 | 1.1000293 |
| Simsek (2013) | 0.9443143 | 0.79959172 | 1.1152309 |
| Tkacova (2005) | 0.95185673 | 0.80476516 | 1.125833 |
| Ulasli (2013) | 0.9390285 | 0.79571593 | 1.1081524 |
| Van Suylen (1999) | 0.93581122 | 0.79111809 | 1.1069683 |
| Wang (2000) | 0.93123245 | 0.78920233 | 1.0988233 |
| Wu (2019) | 0.95072764 | 0.80196643 | 1.1270834 |
| Yildiz (2003) | 0.92097926 | 0.7800957 | 1.087306 |
| Zhang (2008) | 0.93297452 | 0.78889048 | 1.1033744 |
| Combined | 0.93236726 | 0.79113705 | 1.0988093 |

**Table S9 Results of Begg's Test and Egger's Test to detect ID vs. II bias in different populations**

| Ethnicity | n | Begg's | | Begg's | | cont. corr. | | Egger's | |
| --- | --- | --- | --- | --- | --- | --- | --- | --- | --- |
|  |  | score | s.d. | z | p | z | p | bias | p |
| Asian | 8 | 10 | 8.083 | 1.24 | 0.216 | 1.11 | 0.266 | 0.54 | 0.652 |
| Caucasian | 12 | -12 | 14.583 | -0.82 | 0.411 | 0.75 | 0.451 | -0.55 | 0.494 |
| overall | 20 | -2 | 16.673 | -0.12 | 0.905 | -0.06 | 0.952 | -0.20 | 0.750 |

**Table S10 Influence analysis results of DD+ID vs. II**

| Study omitted | Estimate | 95% Conf. Interval | |
| --- | --- | --- | --- |
| Ahsan (2004) | 1.0708944 | 0.91838431 | 1.2487305 |
| Ayada (2014) | 1.0757315 | 0.92294788 | 1.2538067 |
| Busquets (2007) | 1.0439148 | 0.89469033 | 1.2180282 |
| Chen (2014) | 1.0479748 | 0.88990718 | 1.2341188 |
| Gu (2003) | 1.0351427 | 0.88374615 | 1.2124752 |
| Hopkinson (2004) | 1.0722735 | 0.91694951 | 1.253908 |
| Jiang (2002) | 1.0420491 | 0.89396948 | 1.2146571 |
| Kirtipal (2019) | 1.0490494 | 0.89442253 | 1.230408 |
| Kuzubova (2013) | 1.0737854 | 0.91958493 | 1.2538429 |
| Marushchak (2019) | 1.0693388 | 0.91800684 | 1.2456176 |
| Mlak (2016) | 1.0606697 | 0.90407252 | 1.2443913 |
| Pabst (2009) | 1.0929992 | 0.93231833 | 1.2813728 |
| Simsek (2013) | 1.0725015 | 0.91983968 | 1.2505001 |
| Tkacova (2005) | 1.0879735 | 0.93200099 | 1.2700483 |
| Ulasli (2013) | 1.0836496 | 0.92965204 | 1.2631571 |
| Van Suylen (1999) | 1.0742331 | 0.92013717 | 1.2541354 |
| Wang (2000) | 1.0596271 | 0.90944505 | 1.2346096 |
| Wu (2019) | 1.0895054 | 0.93137616 | 1.274482 |
| Yildiz (2003) | 1.0566406 | 0.90660632 | 1.2315041 |
| Zhang (2008) | 1.0678369 | 0.91477013 | 1.246516 |
| Combined | 1.0662829 | 0.91629928 | 1.2408165 |

**Table S11 Results of Begg's Test and Egger's Test to detect DD+ID vs. II bias in different populations**

| Ethnicity | n | Begg's | | Begg's | | cont. corr. | | Egger's | |
| --- | --- | --- | --- | --- | --- | --- | --- | --- | --- |
|  |  | score | s.d. | z | p | z | p | bias | p |
| Asian | 8 | 4 | 8.083 | 0.49 | 0.621 | 0.37 | 0.711 | 0.71 | 0.523 |
| Caucasian | 12 | -16 | 14.583 | -1.10 | 0.273 | 1.03 | 0.304 | -0.24 | 0.775 |
| overall | 20 | -12 | 16.673 | -0.72 | 0.472 | -0.66 | 0.509 | 0.11 | 0.857 |

**Table S12 Influence analysis results of DD vs. II+ID**

| Study omitted | Estimate | 95% Conf. Interval | |
| --- | --- | --- | --- |
| Ahsan (2004) | 1.3556584 | 1.1677872 | 1.573754 |
| Ayada (2014) | 1.4065163 | 1.2094213 | 1.6357313 |
| Busquets (2007) | 1.3700699 | 1.1754795 | 1.596873 |
| Chen (2014) | 1.361374 | 1.1684093 | 1.5862072 |
| Gu (2003) | 1.2205873 | 1.0454005 | 1.4251316 |
| Hopkinson (2004) | 1.3755351 | 1.1814315 | 1.6015289 |
| Jiang (2002) | 1.3379691 | 1.1524973 | 1.5532887 |
| Kirtipal (2019) | 1.1955711 | 1.0188155 | 1.402992 |
| Kuzubova (2013) | 1.3600497 | 1.1692885 | 1.5819322 |
| Marushchak (2019) | 1.3569375 | 1.1695566 | 1.5743397 |
| Mlak (2016) | 1.4214391 | 1.2149554 | 1.6630149 |
| Pabst (2009) | 1.464825 | 1.2540278 | 1.7110565 |
| Simsek (2013) | 1.3401028 | 1.152833 | 1.5577933 |
| Tkacova (2005) | 1.3616413 | 1.1698499 | 1.5848759 |
| Ulasli (2013) | 1.3921369 | 1.1974025 | 1.6185412 |
| Van Suylen (1999) | 1.3724997 | 1.1786727 | 1.5982007 |
| Wang (2000) | 1.3388199 | 1.1533071 | 1.554173 |
| Wu (2019) | 1.3563055 | 1.1673828 | 1.5758023 |
| Yildiz (2003) | 1.3596097 | 1.1703482 | 1.5794777 |
| Zhang (2008) | 1.3521072 | 1.1639216 | 1.570719 |
| Combined | 1.3543543 | 1.1680457 | 1.57038 |

**Table S13 Results of Begg's Test and Egger's Test to detect DD vs. II+ID bias in different populations**

| Ethnicity | n | Begg's | | Begg's | | cont. corr. | | Egger's | |
| --- | --- | --- | --- | --- | --- | --- | --- | --- | --- |
|  |  | score | s.d. | z | p | z | p | bias | p |
| Asian | 8 | 2 | 8.083 | 0.25 | 0.805 | 0.12 | 0.902 | -1.41 | 0.337 |
| Caucasian | 12 | 10 | 14.583 | 0.69 | 0.493 | 0.62 | 0.537 | 0.64 | 0.571 |
| overall | 20 | 12 | 16.673 | 0.72 | 0.472 | 0.66 | 0.509 | -0.17 | 0.839 |
